# Infrared-Transparent Gold Nanoparticles Converted by Tumors to Infrared Absorbers Cure Tumors in Mice by Photothermal Therapy

**DOI:** 10.1371/journal.pone.0088414

**Published:** 2014-02-10

**Authors:** James F. Hainfeld, Michael J. O'Connor, Ping Lin, Luping Qian, Daniel N. Slatkin, Henry M. Smilowitz

**Affiliations:** 1 Nanoprobes, Incorporated, Yaphank, New York, United States of America; 2 Department of Cell Biology, The University of Connecticut Health Center, Farmington, Connecticut, United States of America; Case Western Reserve University, United States of America

## Abstract

Gold nanoparticles (AuNPs) absorb light and can be used to heat and ablate tumors. The “tissue window” at ∼800 nm (near infrared, NIR) is optimal for best tissue penetration of light. Previously, large, 50–150 nm, gold nanoshells and nanorods that absorb well in the NIR have been used. Small AuNPs that may penetrate tumors better unfortunately barely absorb at 800 nm. We show that small AuNPs conjugated to anti-tumor antibodies are taken up by tumor cells that catalytically aggregate them (by enzyme degradation of antibodies and pH effects), shifting their absorption into the NIR region, thus amplifying their photonic absorption. The AuNPs are NIR transparent until they accumulate in tumor cells, thus reducing background heating in blood and non-targeted cells, increasing specificity, in contrast to constructs that are always NIR-absorptive. Treatment of human squamous cell carcinoma A431 which overexpresses epidermal growth factor receptor (EGFr) in subcutaneous murine xenografts with anti-EGFr antibodies conjugated to 15 nm AuNPs and NIR resulted in complete tumor ablation in most cases with virtually no normal tissue damage. The use of targeted small AuNPs therefore provides a potent new method of selective NIR tumor therapy.

## Introduction

Gold nanoparticles (AuNPs) have interesting electromagnetic wave absorption properties that change with size and shape. Many absorb well in the visible spectrum; for example, 40 nm AuNPs absorb light over 100,000 times more than do ordinary organic dyes [1]. They are commonly used in lateral flow test kits, such as home pregnancy tests, since only a few picomoles of AuNPs are visible to the eye. One might imagine that once targeted to tumors, AuNPs could be used to heat tumors by shining light on them. This effect was demonstrated in vitro using anti-EGFr antibody-targeted 40 nm AuNPs that had an absorption maximum at 530 nm. Irradiation with a 514 nm argon laser led to tumor cell ablation [2]. Unfortunately, ∼500 nm light penetrates tissues poorly, so clinical therapy of most lesions would not be practical [1]–[3]. Although increasing the size of solid gold nanospheres shifts their absorption spectrum toward more penetrating red light, increasing the size to 100 nm only increases the absorption maximum to 550 nm [1]. However, the optimal wavelength to use for best tissue penetration is ∼800 nm (near infrared, NIR) where predominantly hemoglobin absorption is decreasing and water absorption is increasing, forming a “tissue window” of best transmission. Even at this optimal wavelength there is still substantial absorption, with the incident radiation being reduced to 1/10 intensity at ∼2 cm (and 1/100 at 4 cm depth) [4].

Gold nanoshells, constructed with a ∼110 nm silica core and a ∼10 nm thick gold outer layer, were discovered to have absorption maxima ∼800 nm which could be tuned by varying the core and shell sizes [5]. These were directly injected intratumorally into large subcutaneous murine tumors and irradiated with a NIR laser (30 min post injection, 820 nm laser, 4 W/cm^2^, 5-mm spot diameter, <6 min), causing measurable damage compared to controls [6]. Such nanoshells (∼2.1 mg Au/kg) were injected intravenously (iv), NIR laser irradiated (6 hrs post injection, 808 nm, 5.5 mm beam diameter, 4 W/cm^2^, 3 min), and found to eradicate small tumors (∼60 mm^3^) for at least 90 days [7], [8]. Surface temperature during the IR irradiation reached ∼50°C. A subcutaneous mouse prostate tumor model was similarly treated (4 W/cm^2^, 3 min, 810 nm laser) and 93% regression was achieved for very small tumors [9], using surprisingly little gold (∼0.04 mg Au/kg). This technology is being developed by Nanospectra Biosciences, Inc., and is in Phase I clinical trials for superficial head and neck cancers.

Gold nanorods ∼50–100 nm in length were also found to absorb in the NIR in their axial direction. 90 nm rods are more efficient by a factor of ∼10 than 140 nm nanoshells, based on a per volume basis because nanorods, unlike nanopshells, contain no large silica particles [1]. Anti-EGFr antibody was adsorbed to gold nanorods and incubated in vitro with epithelial tumor or non-tumor cells. Irradiation with an 800 nm laser showed that the malignant cells required about half the dose for their thermal ablation compared to control cells [10]. PEG-coated 13×47 nm gold nanorods injected iv (20 mg Au/kg) and irradiated 72 hr later with a 810 nm laser (2 W/cm^2^, 5 min, 1 cm beam diameter) resulted in tumor control for at least 50 days [11]. Tumors were again small (∼55 mm^3^ in volume and ∼3 mm thick).

Tangled aggregates of 44 nm gold nanoparticles with fd-phages (each 1 micron in length) were shown to have NIR absorption and have the advantage of programmable phage peptide display for targeting [12], but the aggregates might be too large for effective in vivo therapy or be immunogenic.

A different approach, described here, is to use small (∼1–15 nm) AuNPs which aggregate in tumors and become NIR-absorptive [13], [14]. Small AuNPs have the potential advantages of better tumor penetration and whole body clearance. AuNPs are like antennas: their size must be matched to the wavelength for best absorption. Small AuNPs (1–15 nm) are poorly matched, but when metal nanoparticles approach each other by less than two diameters they couple electrodynamically and act in concert [15], behaving more like a larger continuous particle [3]. For example, red 10 nm AuNPs become blue when aggregated due to an absorption shift to longer wavelengths. This phenomenon was exploited to detect DNA sequences by placing complementary DNA oligonucleotides on different gold particles which changed color when they hybridized, and single base mismatches could be detected by quantifying the extent of the color shift [16]. Lithographic arrays of gold spots spaced closer and closer resulted in a red absorption shift [17].

Antibody-conjugated AuNPs taken up by cognate cells go into the endosomes and lysosomes [18] and can aggregate if, for example, the targeting antibody is degraded by lysosomal enzymes. Aggregation can also be induced by using the low pH of the endosome (pH 5.5) and lysosome (pH 4–5) [19]. At these pHs, AuNPs carrying, e.g., carboxyl groups, can be protonated, reducing the interparticle repelling negative particle charge and promoting closer approach between AuNPs until attractive van der Walls forces take over and cause aggregation. Other strategies include breaking of pH sensitive bonds (such as Schiff's bases and hydrazones) that can transform AuNPs into forms that aggregate.

Our premise is that small AuNPs, which normally do not absorb in the NIR, become biologically aggregated within tumors, resulting in absorbance amplification and preferential hyperthermia when exposed to NIR. Tumors then act as catalysts that aggregate AuNPs (by low pH environment and proteolysis) and increase NIR absorption and resultant heating, leading to their own destruction. This “bio-nano amplification” strategy has several advantages: a) small AuNPs may better penetrate and load tumors than larger prefabricated NIR-absorbing AuNPs, and b) an additional factor of tumor specificity is gained since the particles in surrounding normal tissue and blood do not heat because the particles are not aggregated and do not absorb. This report describes the demonstration of this approach by using small AuNPs for treatment of tumors in mice.

## Materials and Methods

### Ethics Statement

This study was carried out in strict accordance with the recommendations in the Guide for the Care and Use of Laboratory Animals of the National Institutes of Health. The protocol was approved by the Animal Care Committee of the AALAC-approved University of Connecticut Health Center (UCHC), Center for Laboratory Animal Care (Assurance Identification Number: A2882-17, Protocol 210-622). All treatments were performed under anesthesia, and all efforts made to minimize suffering.

### Preparation of Gold Nanoparticles

Chemicals were purchased from Sigma-Aldrich (St. Louis, MO) unless otherwise noted.

Construct 1: Adsorbed antibody (or albumin): 1 L of water containing 5 mL of 2% HAuCl_4_ was boiled and 30 mL of 1% sodium citrate (w/v) added. After cooling, 20 µg of Erbitux (Construct 1) or BSA was mixed per mL of AuNPs and after 5 min the solution was adjusted to 1% BSA. After 30 min, the particles were purified by centrifugation at 16,000 g for 12 min. and washed with water and adjusted to PBS buffer conditions.

Construct 2: Negatively charged (lipoic) gold preparation for 2.5 mg Au: 50 mL of 15 nm AuNPs were prepared by citrate reduction (as above). 2 mL lipoic acid (0.5 mg/mL in DMSO) and 50 µL dithiobis(succinimidyl propionate) (DSP, 1 mg/mL in DMSO) were mixed and added to the AuNP solution. After 10 min, 2.5 mL of a 3 M NaCl solution was added. The precipitate was spun at 7,000 g for 2 min, the pellet resuspended in 50 mL of water and spun again. The pellet was reconstituted in 1 mL 50 mM phosphate buffer pH 7.5 containing 1 mg of Erbitux or mouse IgG antibody and reacted for 3 hrs. The reaction was quenched with glycine solution (50 uL of 1 M glycine solution adjusted to pH 7.5 with 1 M NaOH). The product was concentrated using a 30 K molecular filter (Millipore, Billerica, MA). The retentate was spun at 16,000 g for 30 minutes to remove excess antibody and reconstituted in PBS, spun again and resuspended in PBS.

Construct 3: Schiff's base 15 nm AuNPs (Construct 3) were prepared as follows for 50 mg Au: 1 L of water containing 5 mL of 2% HAuCl_4_ was boiled and 30 mL of 1% sodium citrate (w/v) added. After cooling, 2.3 mL of 1% glutathione (GSH) and 500 µL of K_2_CO_3_ (4N) were added. After incubation for 30 min, the AuNPs were concentrated and purified via centrifugation at 16 kg. 1 mL of 8% glutaraldehyde was added to 50 OD_520_ of AuNP solution, and incubated overnight at 4°C in 5 mM carbonate buffer (pH 8.5). The AuNP-GSH-glutaraldehyde was concentrated and purified again via centrifugation. 5 µg/(OD*mL AuNP) of Erbitux was added to 50 OD of AuNP solution, and incubated for 2 hours. Excess active groups were blocked with ethanolamine for 1 hour. The sample was purified by centrifugation (16 kg) and resuspended in 1% BSA and 0.7× phosphate buffered saline (PBS, 10 mM phosphate buffer, 140 mM sodium chloride, pH 7.4). Yield (gold): 89%

PEG coated AuNPs: 15 nm AuNPs were prepared by citrate reduction as described above. Each 100 mL of AuNP solution was incubated with 8 mg of mono thiol terminated 2K MW polyethylene glycol (Rapp Polymere, Tübingen, Germany) for 2 hours. AuNPs were purified and concentrated by 3 spins at 16 kg followed by resuspension in PBS.

**Figure 1:**
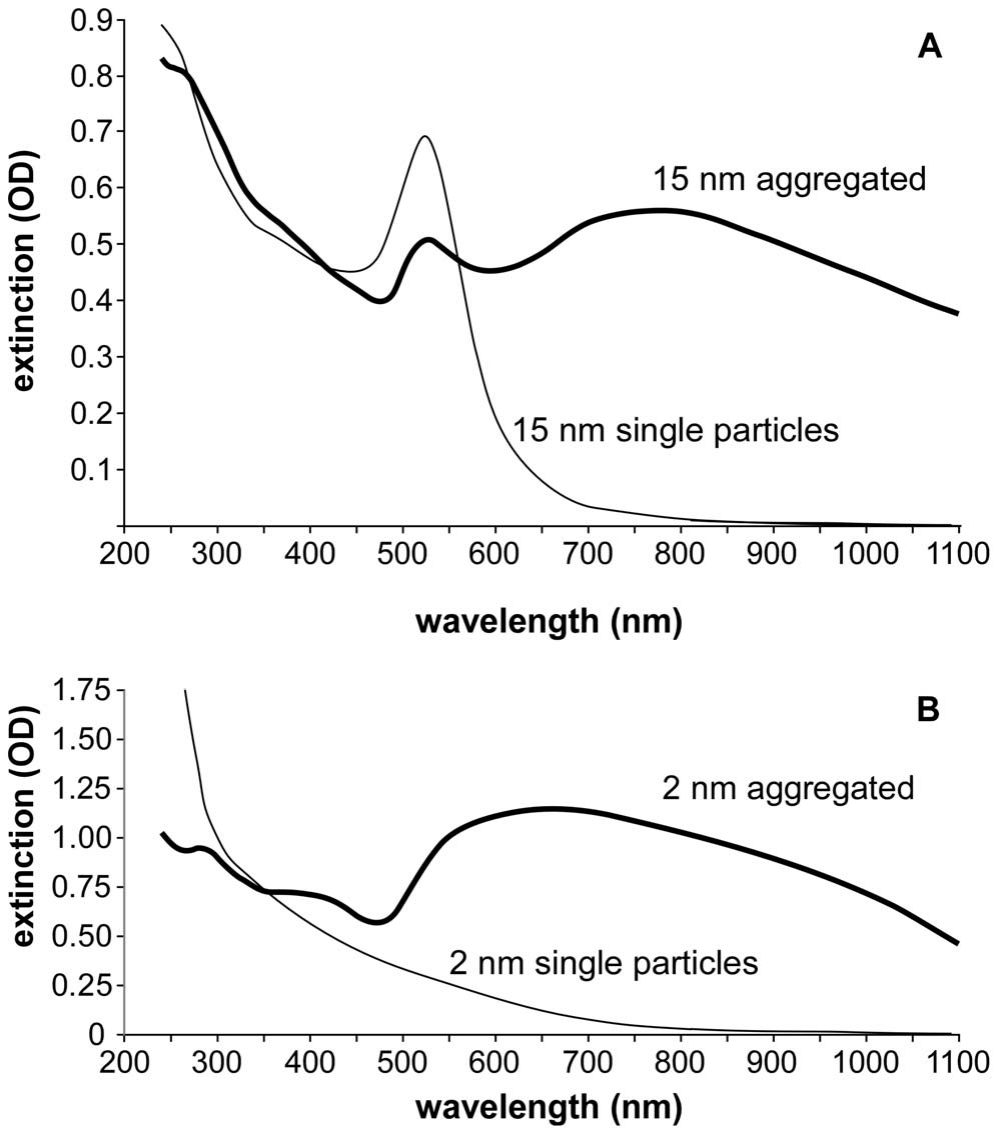
Spectral extinction shift upon aggregation for 15(A) and 2 nm (B) AuNPs. Aggregation was induced by adjustment of pH to 5 in physiologic saline (150 mM NaCl). 15 nm AuNPs were surface modified with lipoic acid, and 2 nm AuNPs had a glutathione shell (which contains two carboxyl groups).

Spectral shift of AuNPs by adjustment of pH to 5 (with 1 N HCl) and 150 mM NaCl (Figure 1) was performed on 15 nm and 2 nm particles 50 mL of 15 nm AuNPs were prepared by citrate reduction (as above). 2 mL lipoic acid (0.5 mg/mL in DMSO) was added to the AuNP solution. 2 nm particles were formed by reacting 25 µL HAuCl_4_ (25 mg/mL) with 30 µL glutathione (10 mg/mL), 13 µL 1 N NaOH and 10 µL sodium borohydride (2 mg/mL) in 1 mL of water. All solutions used water as the solvent.

### Measurement of spectra and dynamic light scattering (DLS)

Spectra and DLS were obtained for AuNP constructs under the various conditions listed in the text.

An Agilent 8452A diode array spectrometer (Agilent Technologies, Inc., Santa Clara CA) was used for UV-Vis measurements, and a Brookhaven Instruments 90 Plus (Brookhaven Instruments Corp, Holtsville, NY) for DLS.

### *In Vitro* AuNP incubation Conditions

Confluent A431 cells derived from an epidermoid carcinoma (ATCC#CRL-1555) were incubated with 5 OD_520_ AuNP solutions in growth medium for times listed in text. Growth medium was DMEM-CM (GIBCO #11995) supplemented with Glutamine (2 mM), Penn/Strep (100 U/mL Pennicillin/100 µg/mL Streptomycin), and Fungizone (0.25 µg/ml), from Invitrogen (Grand Island, NY).

### Mice

One million A431 cells in 25 µL were mixed with 25 µL Maitrigel (BD Biosciences, San Jose, CA) and the mixture was injected subcutaneously in the thighs of female athymic nude mice (Charles River, Kingston, NY). When the tumors reached ∼100–150 mm^3^ the mice were used for the indicated experiments. Tumors were measured at least three times weekly. When tumors reached 500 mm^3^ the mice were euthanized.

### Absorption by aggregated AuNPs in cells measurement

A431 cells in culture were incubated with or without 2 OD_520_ Erbitux-15 nm AuNPs for 2 days, then the culture dishes were washed with PBS. Buffer was removed and dishes were placed vertically in the horizontal beam of a Hewlett Packard 8452A diode array spectrometer. Spectra from 6 separate confluent cell areas were taken.

### Gold analysis of tissues

Tissues were excised, placed in weighed vials, dissolved with aqua regia, and analyzed by graphite furnace atomic absorption spectrometry using a Perkin Elmer 4100Z (Waltham, MA) to determine gold content.

### Measurement of aggregated AuNPs in tissue

Total tissue gold content was measured as described above. A method was devised to measure only the aggregated portion of AuNPs in tissues: Tissue samples below 100 mg were dissolved in 100 µL of 12N NaOH and 100 µL of 20% Triton X-100 in ethanol. Tissues above 100 mg were dissolved in twice the volume of both reagents. Samples were mixed and sonicated at 50 watts for 10 min with ice cooling (larger tissues were sonicated for 20 min). After sonication, two distinct layers of liquid were observed. The solution was vortexed and mixed with 4 times the volume of ethanol to mix the two aqueous layers. Samples were centrifuged for 5 min at 16,000 g. A black pellet at the bottom of the tube was observed after centrifugation. The supernatant was removed and the pellet resuspended in 100 µL of water. Finally, the OD at 800 nm was measured. Tumor and non-tumor values were then compared. This procedure was designed and validated such that unaggregated AuNPs or cells without AuNPs subjected to this procedure did not contribute to the absorption at 800 nm.

### Permeability Measurements

Two 0.7 ml chambers were separated by a 0.4 µm polycarbonate membrane (Millipore, Billerica, MA) [20]. One side was loaded with either 15 nm AuNPs or 10×45 nm Au nanorods (Nanopartz, Loveland, CO) in PBS. Both were stabilized with the same 2,000 MW thio-PEG ligand. The samples were stirred with a magnetic stir bar. The receiving side was loaded with PBS. At various times after starting, the UV-Vis spectrum of the samples was measured to quantify the rate of diffusion. Initial rates were then used to calculate the permeability coeffiecient of the samples according to the equation [21]:
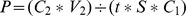



Where P is the solute permeability coefficient, C_2_ is the receiving side concentration, V_2_ is the receiving side volume, t is time, S is the surface area of the membrane, and C_1_ is the starting side concentration.

### NIR heating

A Hydrosun wIRA irradiator, model 09.06.00 (Hydrosun Medizintechnik, GmbH, Germany), was used with a >665 nm pass filter and a 4 mm water filter (water filtered infrared A, “wIRA”). The source was a 150 W halogen lamp. Mice were anesthetized with ketamine (100 mg/kg)/xylazine (8 mg/kg) ip and heated for 1.9 min at 1.5 W/cm^2^.

## Results

Solid spherical AuNPs are virtually transparent at the best wavelength (NIR, 800 nm) for tissue penetration. Although they are colored and absorb in the UV and visible range, there is almost no absorbance in the NIR region. However, aggregation of small AuNPs results in a profound spectral shift into the NIR region (Figure 1). An extinction amplification factor may be calculated by dividing the extinction after aggregation by the extinction before aggregation. At 800 nm, the factor is ∼20, and would lead to increased heating if exposed to 800 nm light.

In order to promote aggregation in tumors, we have exploited five mechanisms: 1) Tumors can be specifically targeted using antibodies to up-regulated growth factor receptors, 2) Antibody-targeted AuNPs are rapidly internalized by receptor mediated endocytosis into tumor cell endosomes and lysosomes [18], 3) Particles displaying carboxyl groups are protonated at the low pH of endosomes/lysosomes which can induce particle aggregation, 4) The low pH can break pH sensitive bonds and dissociate stabilizing moieties, and 5) Lysosomal degradation of the targeting antibody can result in particle aggregation. Many cancers have up-regulated epidermal growth factor receptor (EGFr), so we have used antibodies to this receptor (Erbitux™), and A431 human squamous cell carcinoma cells and tumors which overexpress EGFr.

**Figure 2:**
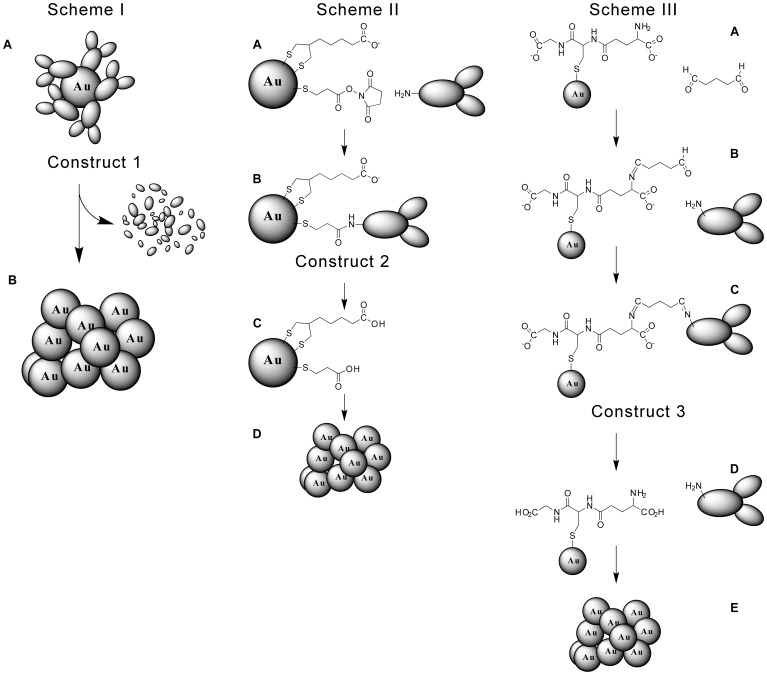
Schemes for tumor delivery and aggregation in endosomes/lysosomes. Scheme 1: AuNPs have anti-tumor antibodies adsorbed (A, Construct 1) that in the lysosome are degraded, allowing the AuNPs to aggregate (B). Scheme II: AuNPs are coated with lipoic acid and dithiosuccinimidyl propionate (DSP) (A), covalently coupled to anti-EGFr antibodies (B, Construct 2), then protonated in the endosome with enzymatic antibody digestion in the lysosome (C), leading to aggregation (D). Scheme III: AuNPs are coated with glutathione (A), reacted with glutaraldehyde (B) and then anti-EGFr antibodies (C, Construct 3) to form Schiff's bases vulnerable to cleavage at endosomal pH (D), resulting in protonation of carboxyl groups and along with enzymatic antibody degredation, causing aggregation (E).

Three constructs were tested: 1) Construct 1 is a 15 nm AuNP with adsorbed anti-EGRr antibodies (Figure 2, Scheme 1). 2) Construct 2 is a 15 nm AuNP with carboxyl groups and covalent attachment of anti-EGRr antibodies (Figure 2, Scheme 2). 3) Construct 3 is a 15 nm AuNP with carboxyl groups and a pH sensitive covalent linkage to the antibody (Figure 2, Scheme 3).

**Figure 3:**
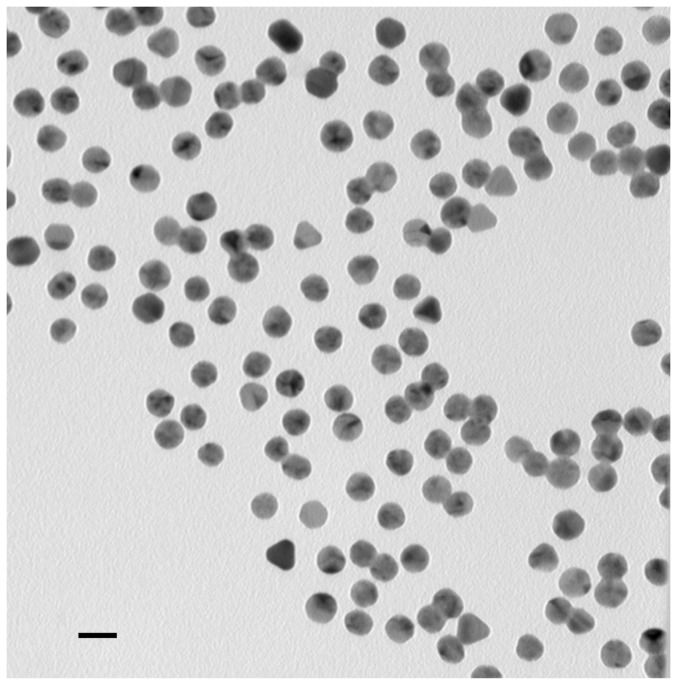
Electron micrograph of Construct 1. Gold core diameter measured 15.3±1.0 nm. Bar  =  20 nm.

The basic properties of the constructs were characterized. An electron micrograph is shown in Figure 3 showing the gold cores to be 15.3 ± 1.0 nm. The hydrodynamic sizes of the constructs were measured by dynamic light scattering (DLS, Table 1). DLS showed a slightly higher value than TEM, similar to other reports of a small size increase due to the citrate layer [22]. The ligand and antibody coatings were seen to increase the size of the particles proportionately.

**Table 1:** Dynamic light scattering size (in nm) of constructs under various conditions (except TEM  =  transmission electron microscope gold core size).

Construct	Starting AuNP	TEM	Ligand Coating	Ligand Coating + Antibody (Erbitux) PBS pH 7.4	90%FBS 37°C	pH 5 37°C 24 hr	Pepsin pH 5 37°C 24 hr
Construct 1	18.8±0.2	15.3±1.0	18.8±0.2	30.7±5.4	28.7±5.9	2004±391	1343±321
Construct 2	18.8±0.2	15.3±1.0	21.6±0.1	38.3±6.0	40.5±8.0	90.8±30.4	1241.0±737
Construct 3	18.8±0.2	15.3±1.0	29.9±10.1	49.2±13.3	47.5±26.3	45.9±9.1	867.7±460

**Figure 4:**
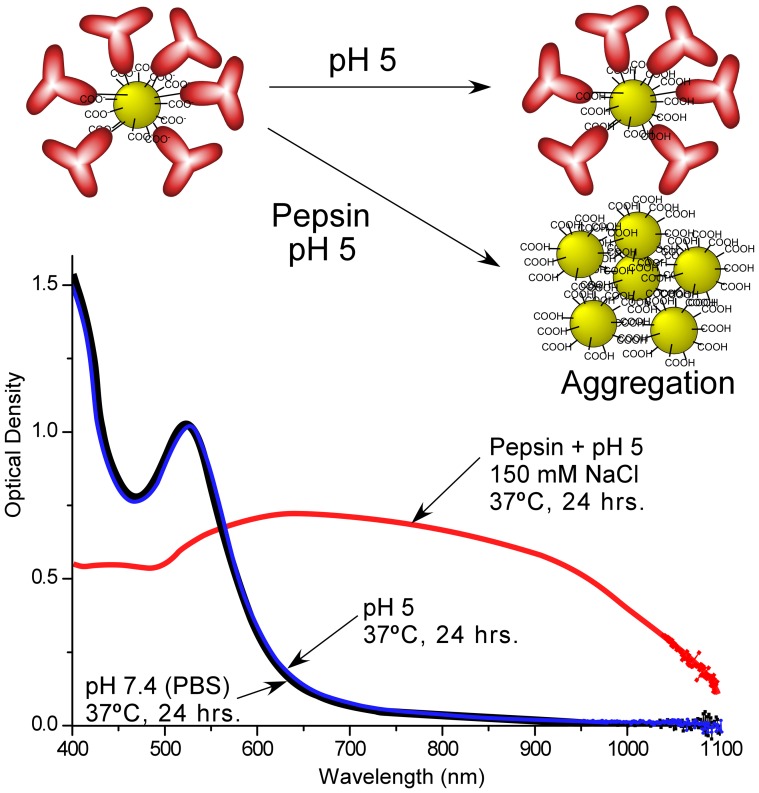
Change in absorption spectra under various conditions. Construct 2 showed stability in PBS at 37°C for 24 hrs (black trace), and also negligible spectral change at pH5 after 24 hrs (blue trace). However, at pH 5 and in the presence of pepsin, extensive aggregation was observed (red trace), greatly increasing the absorbance in the near infrared region.

The constructs should be stable and not aggregate in blood during iv delivery to the tumor. Tests with phosphate buffered saline (PBS), 90% serum, 5% albumin, at 37°C for 24 hrs showed that they did not aggregate under such conditions, as assayed by DLS and UV-Vis spectroscopy (Table 1 and Supporting Information Figures S1, S2, S3, S4, S5, S6 and S7). DLS also showed that exposure to high levels of proteins and serum did not result in measurable adsorption that might enlarge them to the point of losing their small penetrating properties or incurring opsonization and rapid clearance. pH 5 caused Construct 1 to aggregate, but not the other constructs, apparently due to the less stable adsorption vs. covalent conjugation (Figures S8, S9, and S10). However, pH5, 150 mM NaCl, and pepsin (a proteolytic enzyme with peak activity at low pH mimicking lysosomal enzymes) caused all three constructs to heavily aggregate (Table 1 and Figures S11, S12 and S13). A composite diagram of these effects is shown in Figure 4.

**Figure 5:**
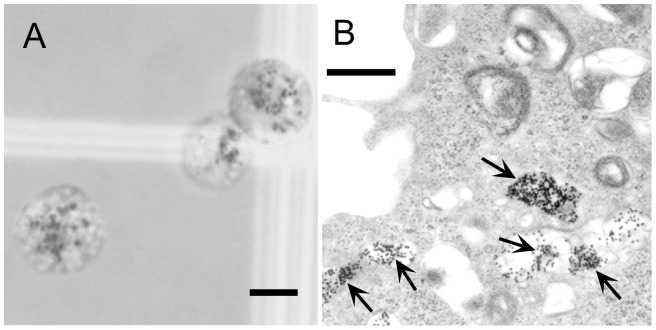
Aggregation of antibody-targeted AuNPs within cells. (A): A431 human epidermoid carcinoma cells with gold feeding (anti-EGFr antibody adsorbed to 15 nm AuNPs, Construct 1) in the growth medium for 3 days. Dark black granules of gold accumulated within the cells. Bar  =  10 µm. (B): Electron micrograph of an A431 cell after only 10 min of incubation with the antibody-AuNPs. There is significant uptake even at this short time into endosomes and aggregation (arrows). Individual 15 nm gold particles may be distinguished as small black dots (bar  =  0.5 µm).

In vitro, Construct 1 AuNPs were found to accumulate in punctate spots in cellular cytoplasm, presumably in endosomes and lysosomes (Figure 5A). Aggregation was apparently enhanced by proteolytic breakdown of the antibody in lysosomes. The localization of AuNPs in endosomes/lysosomes and their aggregation even at an early incubation time (10 min) was evident by electron microscopy (Figure 5B). The gold appears to be non-toxic since cells with AuNPs divided at the same rate as cells without AuNPs.

**Figure 6:**
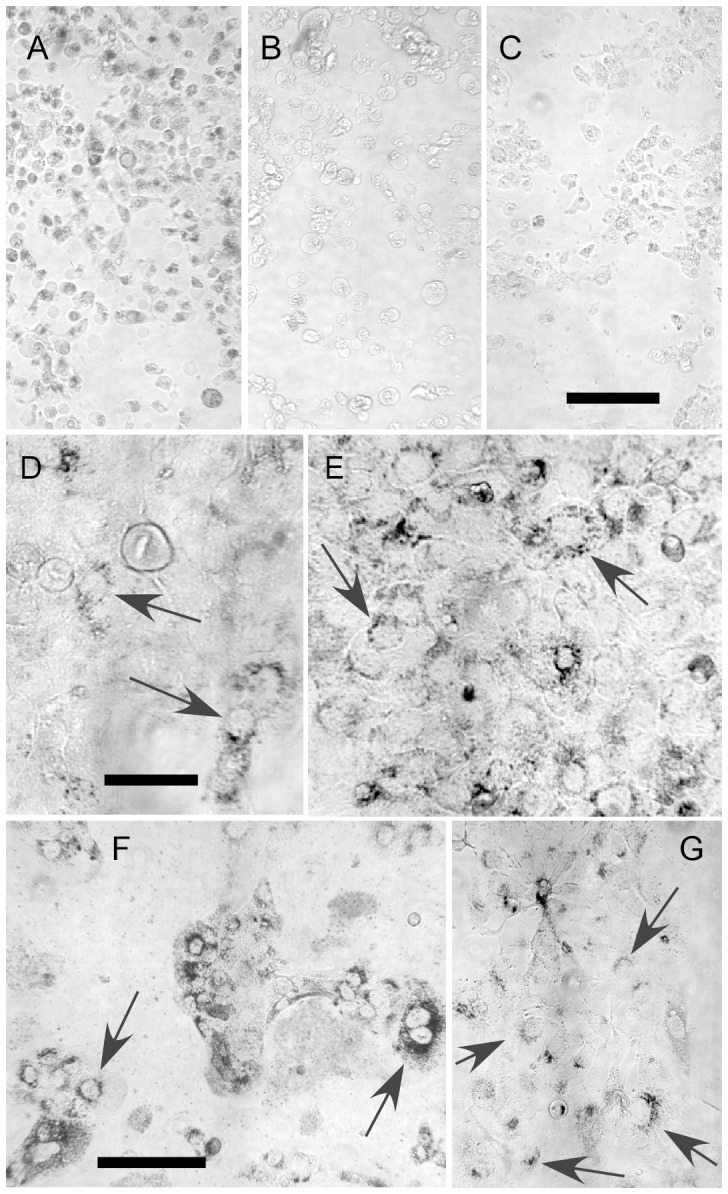
Bright field light micrographs of cells incubated with AuNPs. A–C: A431 human carcinoma cells with AuNP feeding in growth medium for 6 days. 15 nm AuNPs with coatings: (A) lipoic acid with Erbitux linked via DSP (Construct 2, Figure 2); (B) lipoic acid with mouse IgG (non-specific IgG) linked via DSP; (C) AuNPs coated with thio-PEG (2K MW). Only the specific antibody-AuNPs (A) show significant cell uptake. Bar = 100 µm. D,E: Cells with AuNP feeding in growth medium for 2 hours (D), and $ hours (E). The AuNPs are in the cytoplasm as punctate dots indicating residence in endosomes/lysosomes. Their distribution is focused around the outside of the nucleus, but they do not enter it (D, E, arrows). Bar  =  50 µm. (F,G): A431 cells incubated with 15 nm AuNPs which had a surface ligand of glutathione coupled to Erbitux via glutaraldehyde, forming a Schiff's base that is pH sensitive (Construct 3, Figure 2). (F) Cells after 24 hours showing punctate AuNP aggregates in the cytoplasm surrounding, but not inside the nucleus. (G) Cells after 48 hours showing punctate AuNP aggregates capping the nucleus in mostly one region instead of uniformly surrounding it. Bar  =  50 µm.

Construct 2 AuNPs have carboxyl groups which would be more protonated in the acidic environment of the endosome/lysosome, removing negative charge that keeps them apart, allowing them to approach each close enough such that the attractive van der Waals force induces aggregation. Exposure to A431 cells in culture produced obvious cell uptake since the aggregated AuNPs were black in the light microscope (Figure 6A). The effect was also antibody specific since the same construct with mouse IgG (a non-specific antibody) instead of Erbitux showed virtually no cell uptake (Figure 6B). Also, a coating of 2K MW PEG on the AuNPs did not promote uptake into the cells (Figure 6C).

A time course showed that the AuNPs first entered cells and were punctate in the cytoplasm (in endosomes/lysosomes), but tended to accumulate around the nucleus, rather than being homogeneous in the cytoplasm. No AuNP aggregates were seen inside the nucleus. This behavior was observed 2 and 24 hours after the start of incubation (Figure 6D,E).

**Figure 7:**
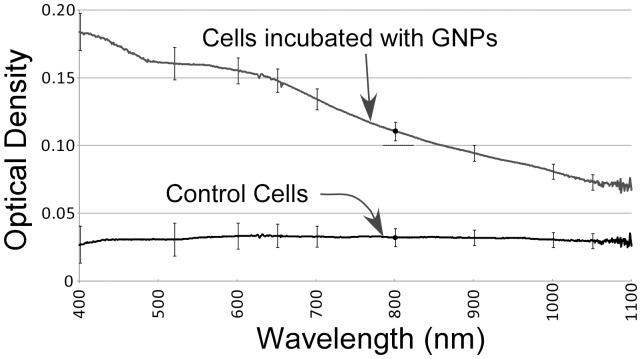
Extinction spectra of cells. Cells incubated with the antibody-AuNP lipoic acid (Construct 2) for 2 days showed absorption at 800 nm compared to control cells without AuNPs, consistent with aggregation of the AuNPs.

Cells incubated with and without Construct 2 were washed in the culture dishes and the extinction of confluent areas measured with a spectrophotometer (Figure 7). The spectra for cells not incubated with gold showed little absorption, consistent with their clear color. The cells incubated with antibody-AuNPs however, showed strong additional absorption, not with the spectrum of isolated AuNPs (Figure 1A), but having substantial extinction at 800 nm, indicative of aggregated AuNPs.

A third approach investigated was to include carboxyl groups on the AuNP surface by using glutathione, and coupling the antibody with a pH labile Schiff's base, again resulting in aggregation at pH 5.5 (Construct 3, Figure 2). Testing of this construct in vitro showed a similar behavior to the lipoic acid construct: The AuNPs entered the target cells and formed punctate aggregates in the endosomes/lysosomes surrounding the nucleus (Figure 6F). At 48 hours, however, most of the AuNPs localized mostly to caps outside and on one side of the nucleus (Figure 6G).

**Figure 8:**
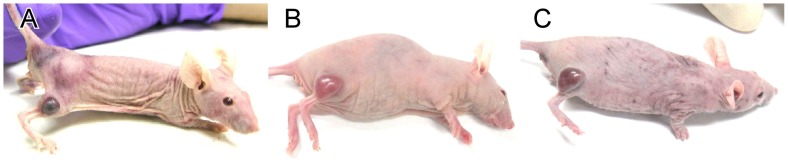
Photographs of mice after intravenous injection of AuNPs. (A): Mouse 1.5 hours after tail vein injection of 15 nm AuNPs with adsorbed anti-tumor antibody (Construct 1) which localized to the tumor on its leg. (B): Mouse before injection of AuNPs. (C): Mouse in (B) 1.5 hours after iv injection of non-targeted 15 nm AuNPs coated with 2k MW PEG.

For in vivo testing, A431 human squamous cell carcinoma cells were subcutaneously implanted into nude mice. The anti-EGFr-AuNP conjugates were injected intravenously via tail vein and showed tumor localization (Figure 8). Mice were dissected and tissues analyzed for gold content by atomic absorption spectroscopy. Six hours post iv injection the tumor to muscle ratio was 12.9±1.8∶1. However, this measurement only indicates the total gold ratio, and does not indicate if the gold is aggregated or not. Aggregated AuNPs were measured by isolating them from tumor and non-tumor (muscle) tissue by a gentle extraction using NaOH and detergent shown to isolate only aggregated AuNPs. This gave a tumor to non-tumor ratio of 19.2∶1.

**Figure 9:**
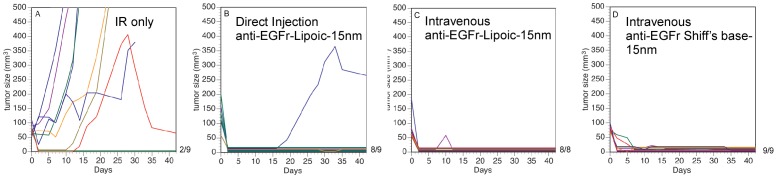
Plots of tumor volume vs. time after IR treatments. (A): IR only, 22% survival (2/9); (B): IR after direct intratumoral injection of 0.2 g Au/kg 15nm-anti-EGFr lipoic acid AuNP preparation (Construct 2), 89% tumor ablation (8/9); (C): IR after iv injection of 1.0 g Au/kg Construct 2, 100% tumor ablation (8/8); (D): IR after iv injection of 1.5 g Au/kg 15 nm-anti-EGFr Schiff's base AuNP preparation (Construct 3), 100% tumor ablation (9/9). IR treatment: 1.5 W/cm^2^, 1.9 min.

**Figure 10:**
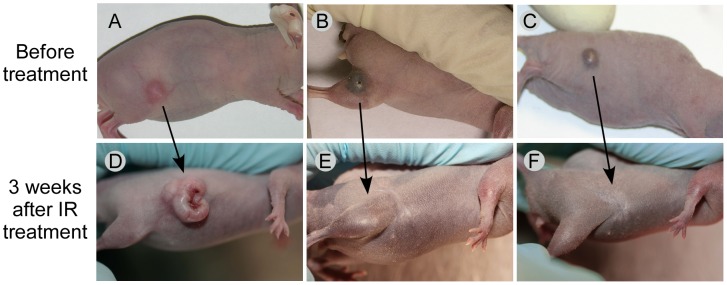
Mice with tumors before and after IR/AuNP treatment. Top row before treatment, bottom row 3 weeks after IR treatment. (A, D): No AuNPs, but exposed to IR. (B,E): Mouse with 10 µL direct intratumoral injection of 15nm-anti-EGFr lipoic acid AuNP preparation (Construct 2) and IR. (C,F): Mouse with intravenous 15nm-anti-EGFr lipoic acid AuNP preparation and IR. There was no residual normal tissue or body impairment with little to no scarring seen with the AuNP-IR treated animals. IR treatment: 1.5 W/cm^2^, 1.9 min.

Constructs 2 and 3 were used to treat mice with tumors using AuNP administration followed by NIR irradiation. A control group with IR exposure alone without any AuNPs resulted in 22% (2/9) tumor ablation (Figure 9A). The lipoic acid preparation (Construct 2) resulted in 89% (8/9) tumor ablation after direct intratumoral injection (Figure 9B), and 100% ablation after intravenous injection and IR exposure (Figure 9C). The Schiff's base preparation (Construct 3) after iv injection and IR exposure resulted in 100% tumor ablation (Figure 9D). Exemplary animals before and after treatments are shown in Figure 10. Animals with ablated tumors exhibited no significant residual scar, no underlying tissue damage, and no functional disability (Figure 10E,F).

Many studies validate that smaller nanoparticles penetrate tissues and tumors better than larger ones [23]–[26], but we conducted additional experiments to confirm this. A diffusion cell was set up to measure the transport of 15 nm AuNPs or 10×45 Au nanorods through a separating porous membrane [20]. Both were PBS-stabilized with the same 2,000 MW thio-PEG ligand. The 15 nm particles had a permeability coefficient of 1.83×10^−3^ cm/min whereas the nanorods had a permeability coefficient of 1.01×10^−3^, showing the smaller 15 nm spherical AuNPs penetrate at a rate 1.81 times that of NIR-absorbing gold nanorods.

## Discussion

Reported here is the use of small AuNPs that are essentially transparent to 800 nm light that are actively converted by tumor cells into highly absorbing particles. The cells essentially act as catalysts for this conversion, which then leads to amplification of absorption when exposed to NIR, increased heating, and cell self-destruction. This differs from the nanoshell and nanorod approach where larger AuNPs structures are used that are prefabricated to absorb strongly at 800 nm. The small particle strategy may offer advantages over larger particles: a) better tumor penetration due to their smaller size (Figure S14), and b) better tumor-to-non-tumor heating ratios since background AuNPs in non-tumor tissue that are not taken up by cells essentially do not absorb. The latter could provide a significant benefit since limiting damage to surrounding normal tissue can be crucial and could allow a larger treatment dose to the tumor to be applied. For example, if targeted nanoparticles have a 8∶1 tumor-to-surrounding non-tumor biodistribution, one would normally expect a treatment ratio (tumor:non-tumor) of 8∶1. However, if the nanoparticles in the tumor are also 10 times more absorbing than those in the non-tumor, then the treatment ratio becomes 80∶1. Such tumor specificity is rarely achieved by other means.

AuNPs are endocytosed by cells, particularly if attached to a receptor ligand such as an internalized antibody, and accumulate in endosomes/lysosomes [18]. To maximize their close-range aggregation for maximal absorptive shift into the NIR region, it is desirable to: a) maximize their uptake and b) ensure that they closely aggregate. Tumor specificity is important, so an obvious design is to include an antibody to the tumor (or peptide or other targeting molecule) that undergoes receptor-mediated endocytosis. Once in the endosome, aggregation can be optimized by using chemical groups that promote aggregation and using small ligands that permit close distances between the aggregated particles, thus maximizing NIR absorption. Peptide linkers could be used that are substrates for lysosomal enzymes or tumor enzymes (e.g., metalloproteinases). Another approach is to use covalent or non-covalent bonds that are pH sensitive and are broken at the ∼5.5 pH of the endosome and <5 in the lysosome [27]. A number of covalent bonds fall into this category, including esters, diortho esters, Schiff's bases, cyclic anhydrides, such as maleic or citraconic anhydrides, acetal bonds, β-thiopropionate, and hydrzones [27]. Intracellular reducing power (e.g., glutathione and thiol concentration) is higher compared to that found extracellularly, and may be used to remove components from the AuNP shell [28] and expose groups that promote aggregation. Non-covalent components can also be disrupted by a change in pH, typically the association of charged groups. A simple design is the use of a carboxylated AuNP ligand shell that upon protonation at pH 5 reduces the charge repulsion between AuNPs and induces aggregation. The amount and size of aggregates might possibly be controlled by varying the concentration of AuNPs applied, the total dose of AuNPs administered, varying the targeting ligand (which would modulate receptor-mediated endocytosis), as well as the varying the AuNP ligand driving aggregation (e.g., more carboxyl groups and smaller ligand size for tighter packing).

The average tumor size we used in this study was 84 mm^3^ which is several times larger than those reported for nanoshells and nanorods (e.g., ∼3 mm diameter, ∼27 mm^3^). One must be careful in this hyperthermia approach since tumor cells can be more sensitive to heat than normal tissue [29], [30] and from our experience it is even possible to cure some small tumors with NIR alone (Figure 9A). The addition of a small amount of absorbing nanoparticles may tip the balance from not being eradicated to full ablation. This may explain why significantly lower gold doses are reported for the nanoshell treatments (∼0.04–2 mg Au/kg). More gold was used for the nanorod treatment study (20 mg Au/kg), but still less than the amount we have used in our study (1 g Au/kg). We only used high levels to demonstrate proof-of-principle, and have not yet investigated what the lowest dose of small AuNPs is needed to be effective, and therefore it could be in the range of some of these other studies, or perhaps even lower. Of course, lower doses are preferable for reducing potential toxicity and whole body retention.

Although lasers, e.g., at 800 nm, are good sources for NIR therapy, it is shown here that a simple halogen lamp (replacement cost ∼$12) with a simple water (low pass) and high pass filters can be used to provide effective treatment. A spectrum is shown in Figure S15. Water filtered infrared A (wIRA) lamps are commonly used clinically in wound healing [31]–[34] and treatment of warts [35]. They are readily and inexpensively available, and do not require extensive safety regulation. Because the gold absorption is not strictly only at 800 nm, a wider spectral range may even be more efficient than monochromatic 800 nm light from a laser.

The combination of hyperthermia with radiation or chemotherapy has been shown to be synergistic [36]–[38], but difficult to implement clinically in many cases, and therefore not routinely used. NIR irradiation of tumors containing AuNPs for generating local tumor heating could provide a method to realize this synergy for appropriate tumors. Although our results are dramatic in mice, what are the obstacles for clinical translation? The use of gold may incur more cost than some other medications. Also, unless nanoparticles are very small (<5 nm) or broken down, they do not exit via the kidneys and excretion via feces is much slower. Many nanoparticles are taken up by non-tumor cells and may be retained for months or longer. Since many nanoparticles are colored, the skin can be discolored for long periods, which, at some level, may be cosmetically objectionable. Although many forms of gold nanoparticles appear to be non-toxic [39]–[41], more thorough toxicity studies are needed. One limitation of this AuNP-NIR approach is the poor penetration of light in tissue. At 800 nm, the optimal “tissue window” where absorbance is minimal, the intensity is only 10% of the incident intensity at 2 cm [4]. Loading with AuNPs may even make this worse due to their own absorption. Fiber optic light pipes with diffusers have been proposed to insert into deeper tumors, but this approach is invasive and assumes that the irregular shapes of tumors are known and the multiple light pipes be adequately placed. Nevertheless, the approach might first be applicable to superficial tumors on the skin or in the head and neck region. Small AuNPs might offer an advantage due to the poor penetration of large AuNPs into tumors. It was shown that albumin (7 nm, 68 kDa) leaked into tumors but 100 nm liposomes did not [23], thus limiting penetration of large nanoparticle constructs. A further worry is the poor clearance of AuNPs in this large size range due to long-term retention in the liver, spleen and other tissues. Direct intratumoral injection may be useful for some tumors, but suffers from invasiveness and need for precise injections that typically do not adequately cover irregular and large tumors. Nevertheless tumor control by IR after direct gold injection was impressive (Figure 9B). Deeper tumors might be accessible via laparoscopy, catheterization, or surgery. Retreatments of thicker tumors may also be possible.

## Conclusion

We have shown that tumor cells can effect the aggregation of small gold nanoparticles and shift their absorption into the near infrared region. By designing the organic shell, these gold nanoparticles may be targeted to tumor cells in vivo and become substrates for tumor-specific cellular conversion into highly absorbing aggregates. Application of near infrared light resulted in substantial tumor ablation in mice with sparing of normal tissues. This approach holds promise for clinical application to tumors accessible to 800 nm light.

## Supporting Information

Figure S1 **AuNP Construct I incubated with 90% (by volume) serum (FBS) for 24 hours showed little change from the starting AuNP construct spectrum, with no increase in absorbance in the NIR or shift in the surface plasmon peak.** Construct I (black), Construct I in serum for 24 hrs at 37°C (red).
(TIF)

Figure S2 **AuNP Construct 2 incubated with serum (FBS) for 24 hours showed little change from the starting AuNP construct spectrum, with no increase in absorbance at 800 nm.** Construct 2 (black), Construct 2 in 90% serum (by volume) for 24 hrs at 37°C, then centrifuged and AuNPs resuspended.
(TIF)

Figure S3 **AuNP Construct 3 incubated with serum for 24 hours showed little change from the starting AuNP construct spectrum, with no increase in absorbance at 800 nm.** FBS (red); FBS plus Construct 3 (black); FBS plus Construct 3 minus FBS control (blue); Construct 3I (green).
(TIF)

Figure S4 Construct 1 incubated with 5% BSA in PBS at pH 7.4, 37°C for 24 hrs (red) showed virtually no increased absorbance in the NIR region and no shift in the gold surface plasmon resonance peak. Shown are spectra for the original Construct I in PBS (black) and 5% BSA (blue).
(TIF)

Figure S5 **Construct I (black) incubated with 5% BSA, PBS, pH 7.4, 37**°**C for 1 hr, then purified by centrifugation and resuspended in PBS (red).** No change in aggregation was apparent.
(TIF)

Figure S6 **UV-Vis spectra of Construct 2 incubated with 5% BSA in PBS at pH 7.4, 37**°**C for 24 hrs (red) showed virtually no increased absorbance in the NIR region and no shift in the gold surface plasmon resonance peak compared to the construct without 5% BSA (black).**
(TIF)

Figure S7 **Spectra of Construct 3 before (black spectrum) and after (red spectrum) incubation with 5% BSA for 60 hrs at 37**°**C.At 60 hrs, the AuNPs were centrifugally purified from BSA and resuspended in PBS.** No alteration in the AuNP spectrum was observed.
(TIF)

Figure S8 **Incubation of Construct I with either PBS (black) or 50 mM phosphate buffer, pH 5 (red) at 37**°**C for 24 hrs.** The low pH caused a red shift of the spectral peak and considerable more absorption in the NIR region.
(TIF)

Figure S9 **Spectra of Construct 2 incubated at pH 5 for 24 hrs at 37**°**C (red) showed no change from incubation at pH 7.4 (black).**
(TIF)

Figure S10 **Spectra of Construct 3 incubated at pH 5 for 24 hrs at 37**°**C (red) showed slight change from incubation at pH 7.4 (black).**
(TIF)

Figure S11 **Shift of absorption into the NIR region when Construct 1 (black) was exposed to pepsin (red) for 24 hrs at pH 5.**
(TIF)

Figure S12 **Shift of absorption into the NIR region when Construct 2 (black) was exposed to pepsin (red) for 24 hrs at pH 5.**
(TIF)

Figure S13 **Shift of absorption into the NIR region when Construct 3 (black) was exposed to pepsin (red) for 24 hrs at pH 5.**
(TIF)

Figure S14 **Size comparison of gold nanoshells, nanorods, 15 and 2 nm gold particles and an IgG molecule.** The smaller ones may have substantially better diffusion, tumor access, and clearance properties.
(TIF)

Figure S15 **Water filtered infrared A (wIRA) lamp spectrum used with 665 nm pass filter.**
(TIF)

File S1 **Supporting Information.**
(DOCX)
