# Supplementary figures and images for: Infrared-Transparent Gold Nanoparticles Converted by Tumors to Infrared Absorbers Cure Tumors in Mice by Photothermal Therapy
Source: PLoS One. 2014 Feb 10;9(2):e88414. doi: 10.1371/journal.pone.0088414 (PMC3919775; doi:10.1371/journal.pone.0088414)

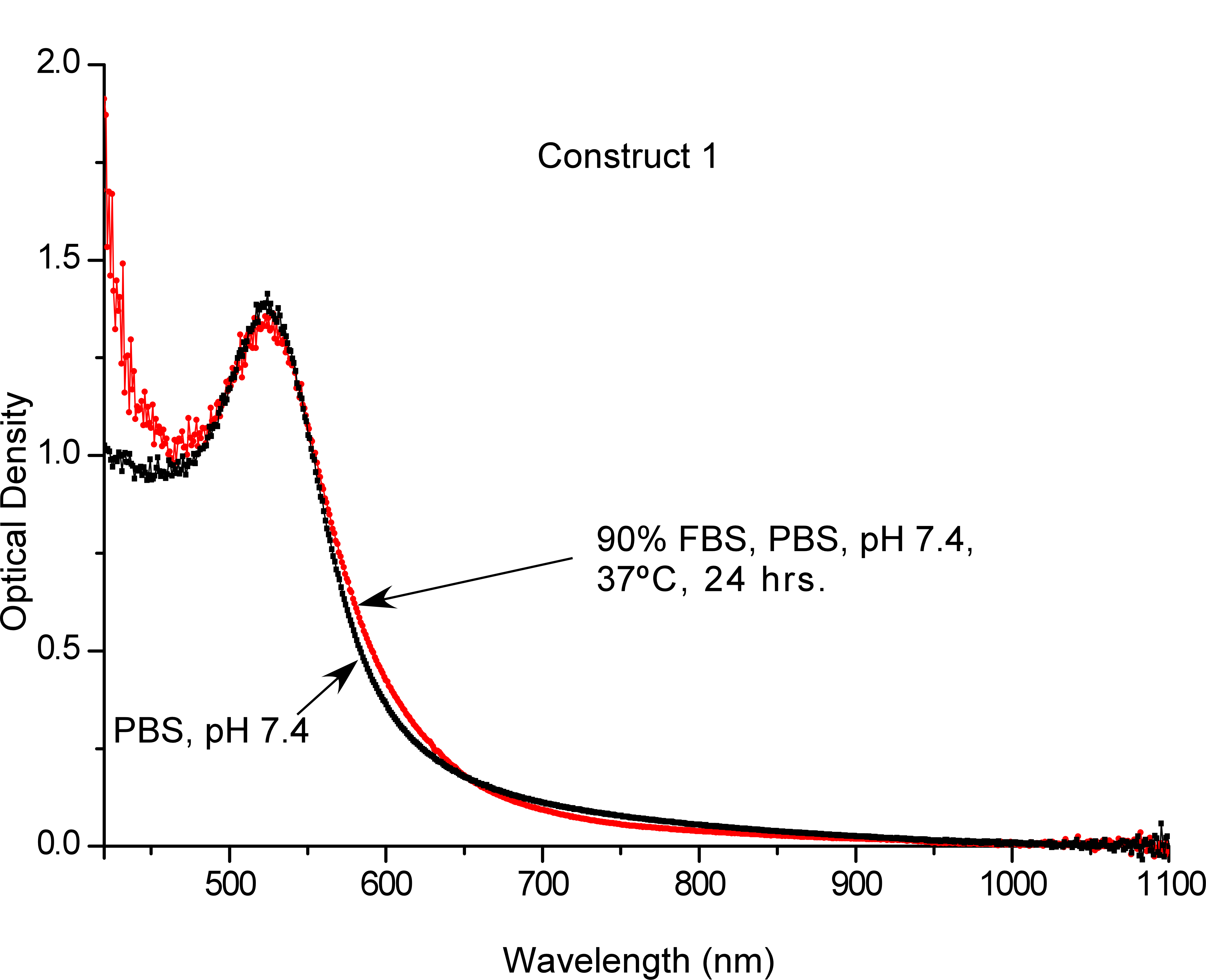

Supplement: Figure S1 — AuNP Construct I incubated with 90% (by volume) serum (FBS) for 24 hours showed little change from the starting AuNP construct spectrum, with no increase in absorbance in the NIR or shift in the surface plasmon peak. Construct I (black), Construct I in serum for 24 hrs at 37°C (red). (TIF) [file pone.0088414.s001.tif]

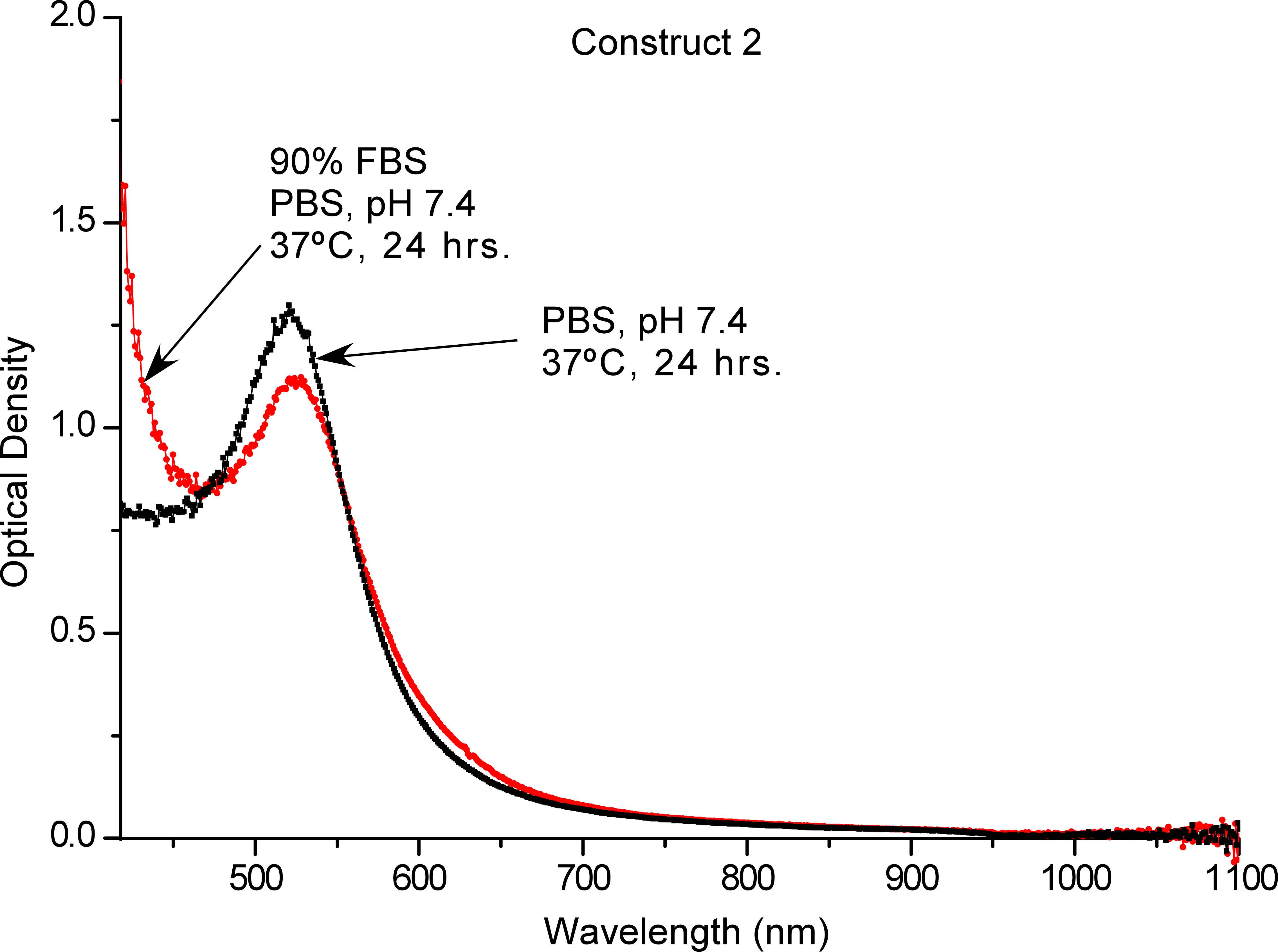

Supplement: Figure S2 — AuNP Construct 2 incubated with serum (FBS) for 24 hours showed little change from the starting AuNP construct spectrum, with no increase in absorbance at 800 nm. Construct 2 (black), Construct 2 in 90% serum (by volume) for 24 hrs at 37°C, then centrifuged and AuNPs resuspended. (TIF) [file pone.0088414.s002.tif]

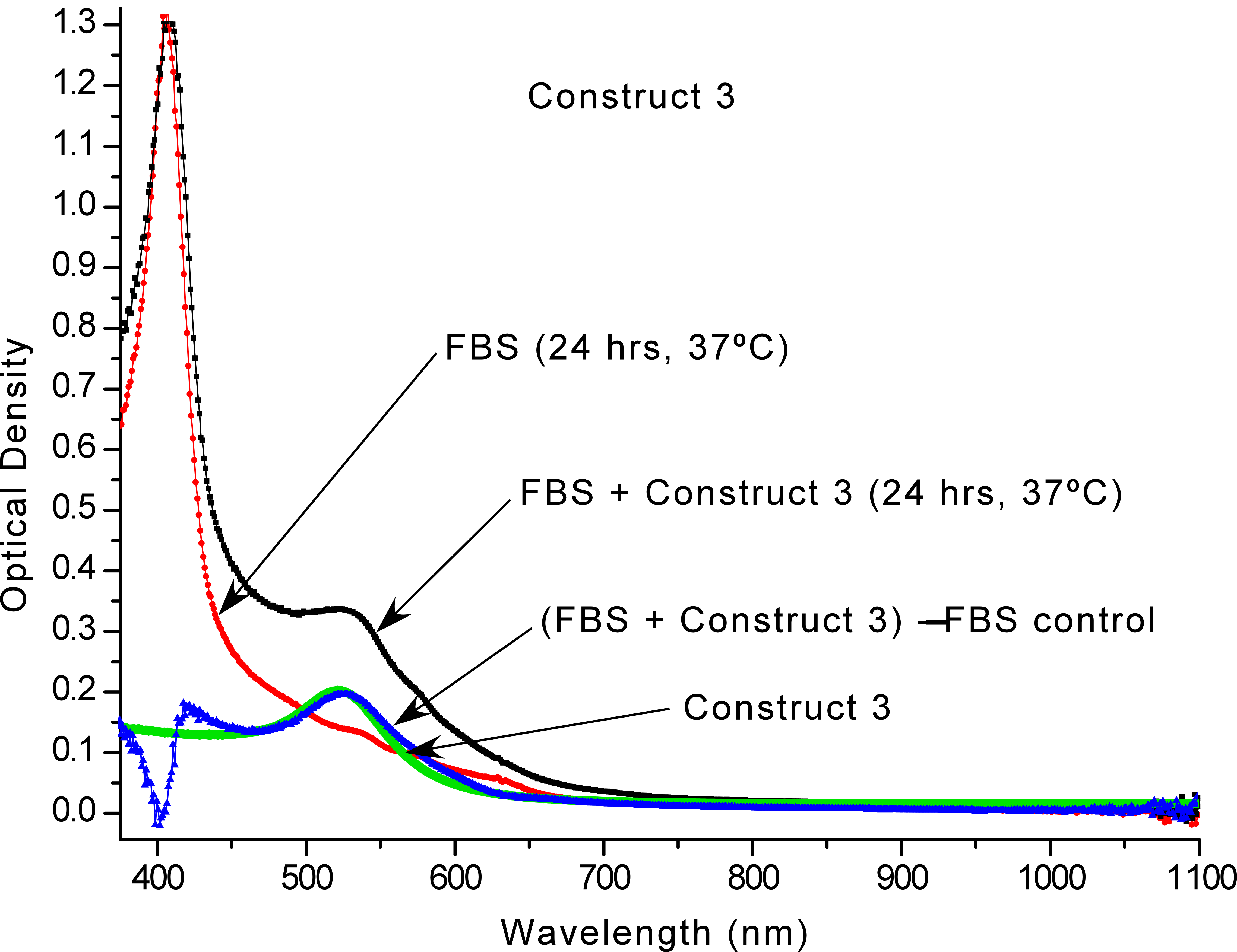

Supplement: Figure S3 — AuNP Construct 3 incubated with serum for 24 hours showed little change from the starting AuNP construct spectrum, with no increase in absorbance at 800 nm. FBS (red); FBS plus Construct 3 (black); FBS plus Construct 3 minus FBS control (blue); Construct 3I (green). (TIF) [file pone.0088414.s003.tif]

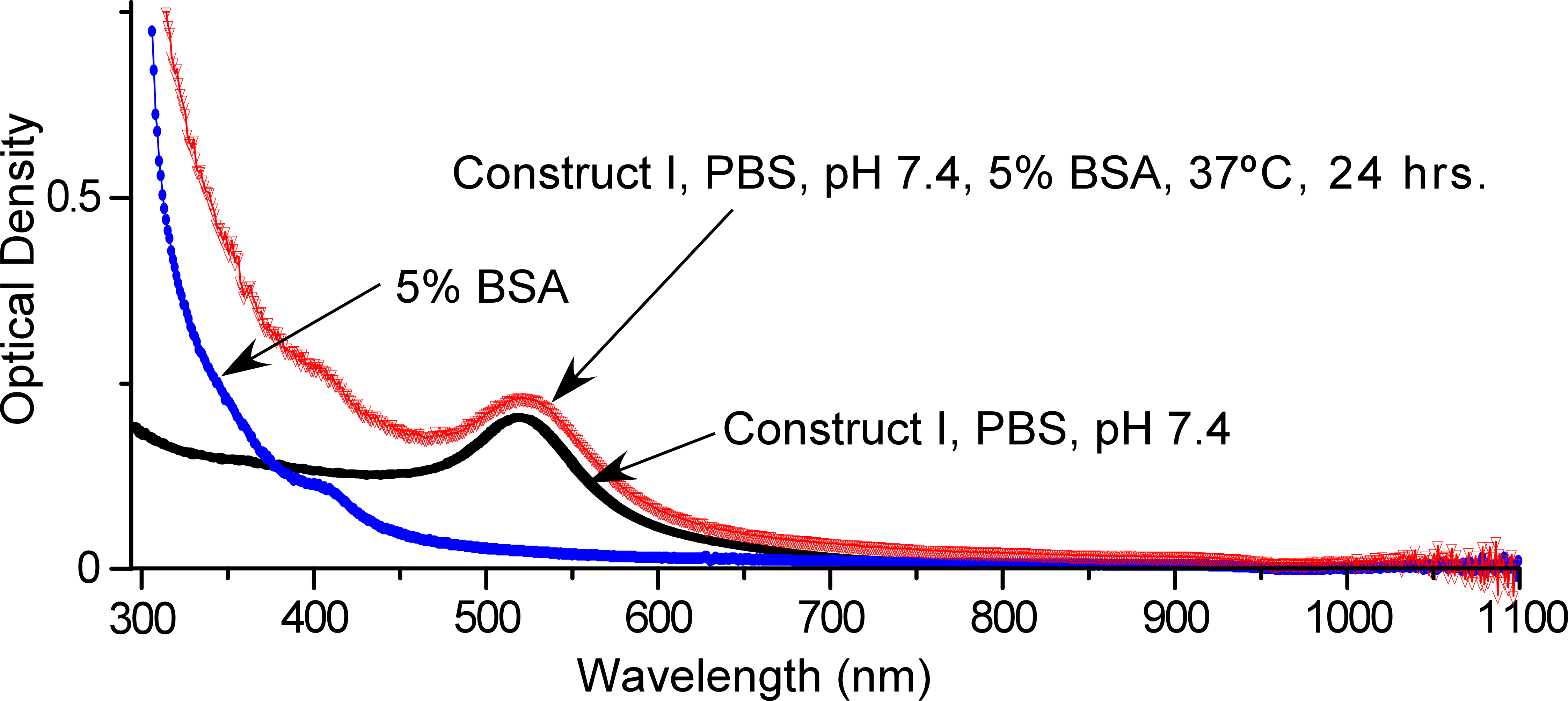

Supplement: Figure S4 — Construct 1 incubated with 5% BSA in PBS at pH 7.4, 37°C for 24 hrs (red) showed virtually no increased absorbance in the NIR region and no shift in the gold surface plasmon resonance peak. Shown are spectra for the original Construct I in PBS (black) and 5% BSA (blue). (TIF) [file pone.0088414.s004.tif]

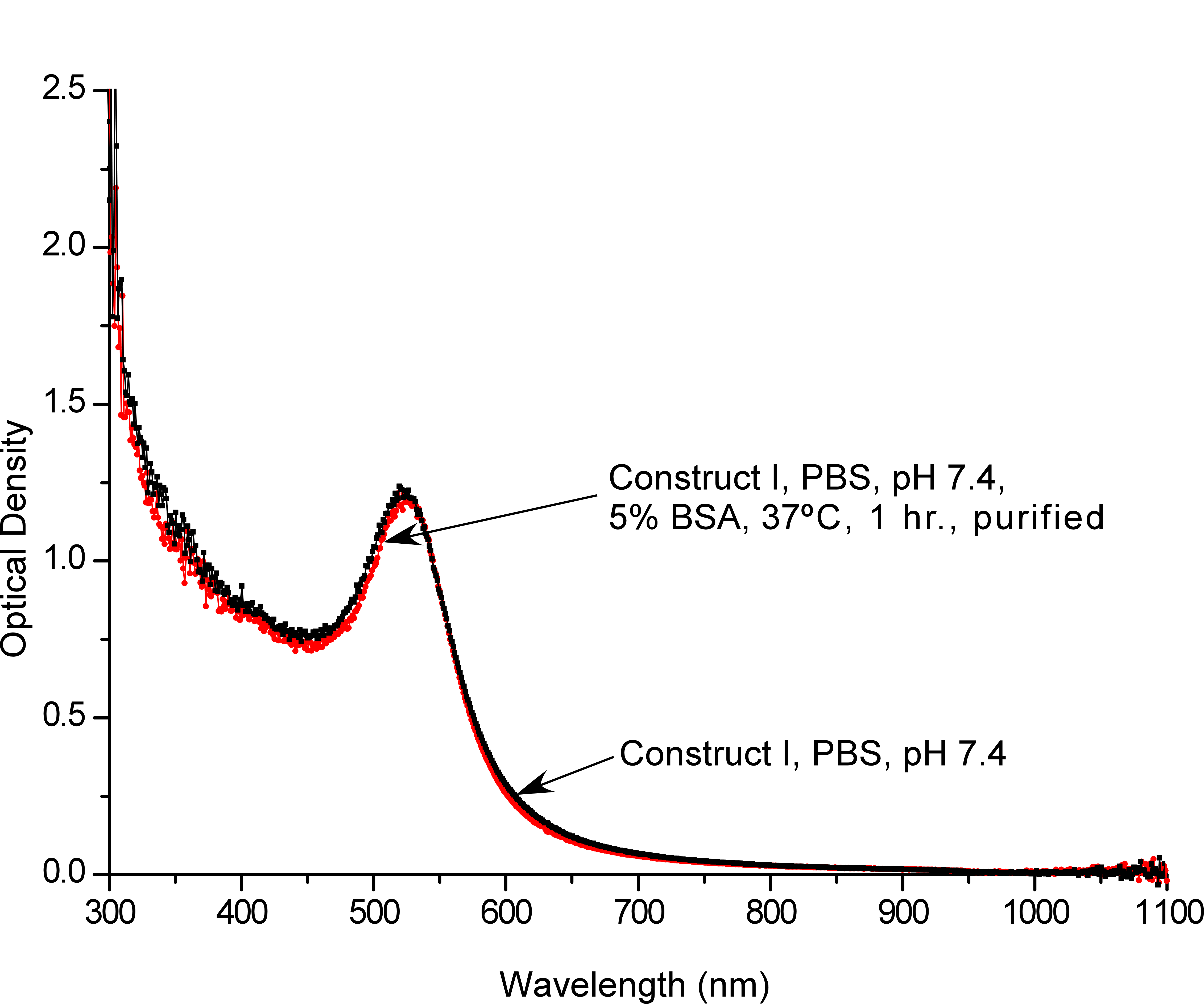

Supplement: Figure S5 — Construct I (black) incubated with 5% BSA, PBS, pH 7.4, 37°C for 1 hr, then purified by centrifugation and resuspended in PBS (red). No change in aggregation was apparent. (TIF) [file pone.0088414.s005.tif]

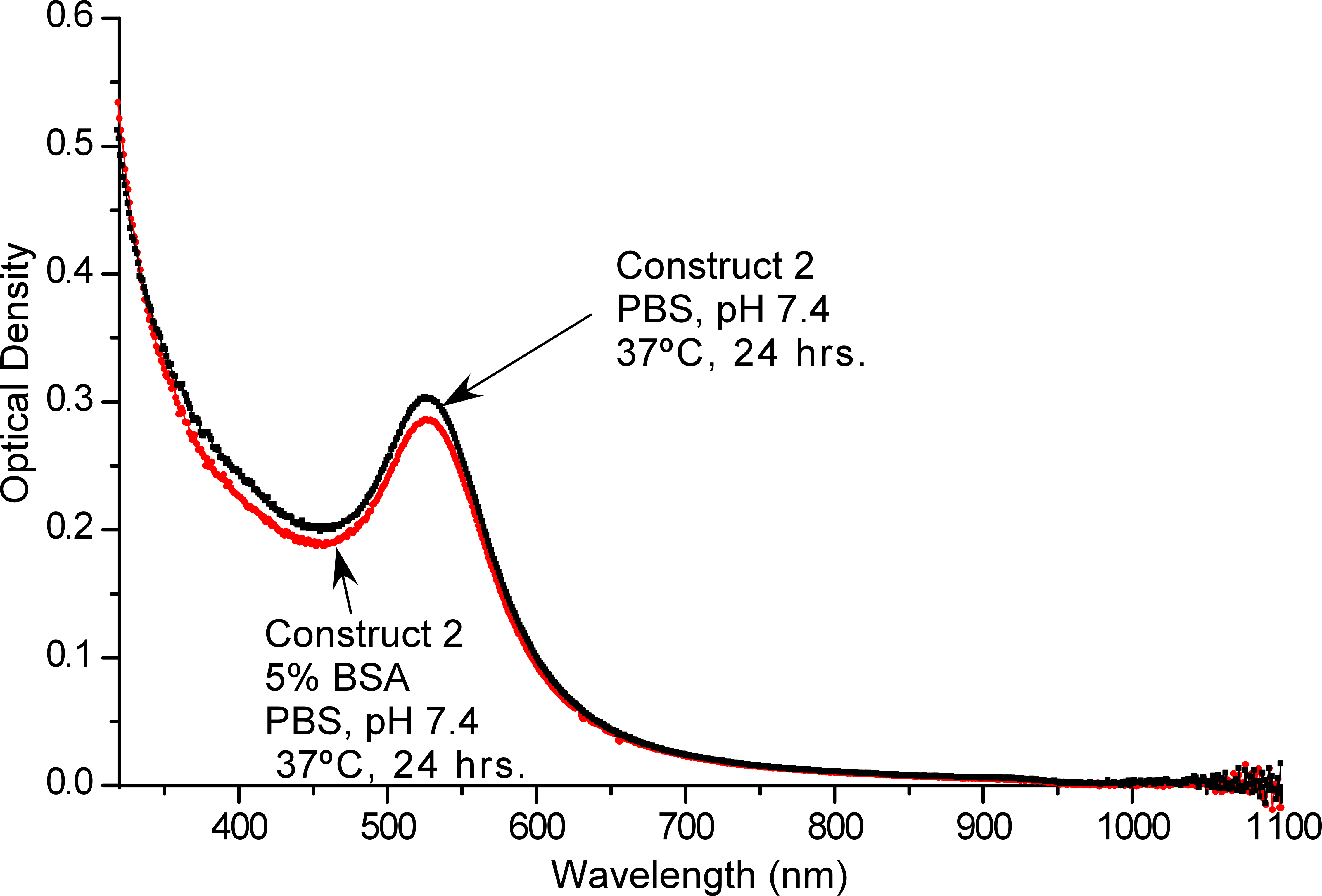

Supplement: Figure S6 — UV-Vis spectra of Construct 2 incubated with 5% BSA in PBS at pH 7.4, 37°C for 24 hrs (red) showed virtually no increased absorbance in the NIR region and no shift in the gold surface plasmon resonance peak compared to the construct without 5% BSA (black). (TIF) [file pone.0088414.s006.tif]

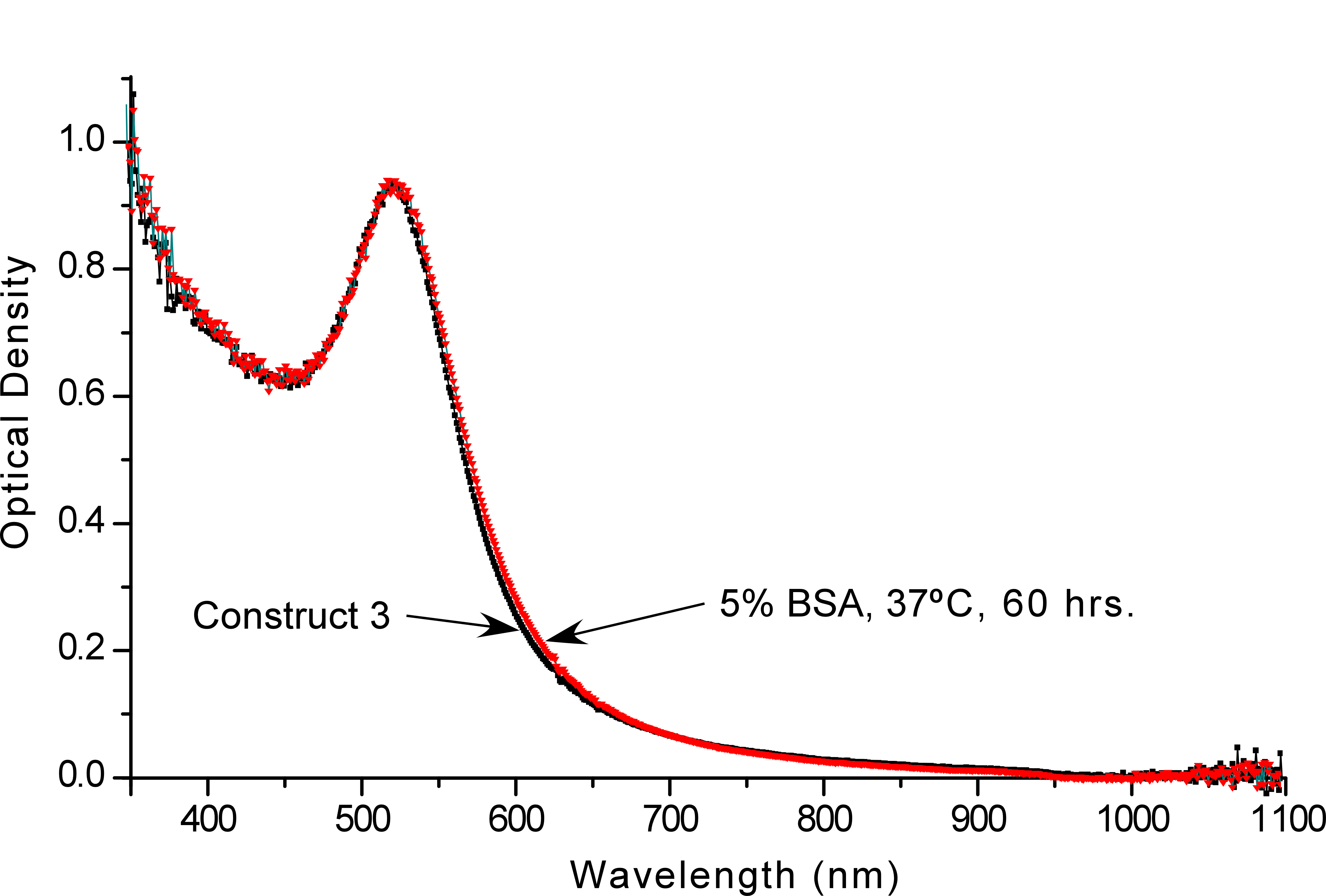

Supplement: Figure S7 — Spectra of Construct 3 before (black spectrum) and after (red spectrum) incubation with 5% BSA for 60 hrs at 37°C.At 60 hrs, the AuNPs were centrifugally purified from BSA and resuspended in PBS. No alteration in the AuNP spectrum was observed. (TIF) [file pone.0088414.s007.tif]

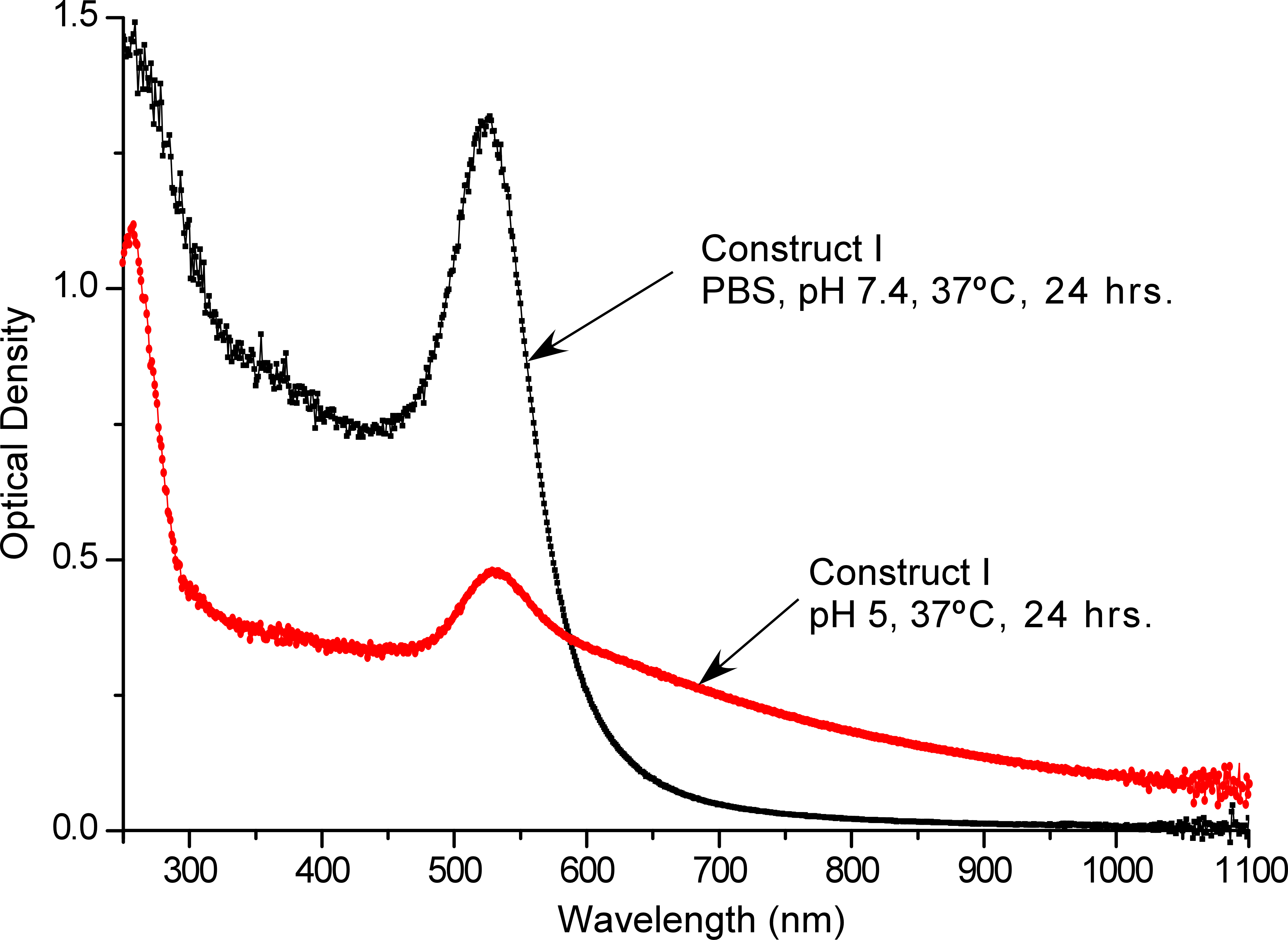

Supplement: Figure S8 — Incubation of Construct I with either PBS (black) or 50 mM phosphate buffer, pH 5 (red) at 37°C for 24 hrs. The low pH caused a red shift of the spectral peak and considerable more absorption in the NIR region. (TIF) [file pone.0088414.s008.tif]

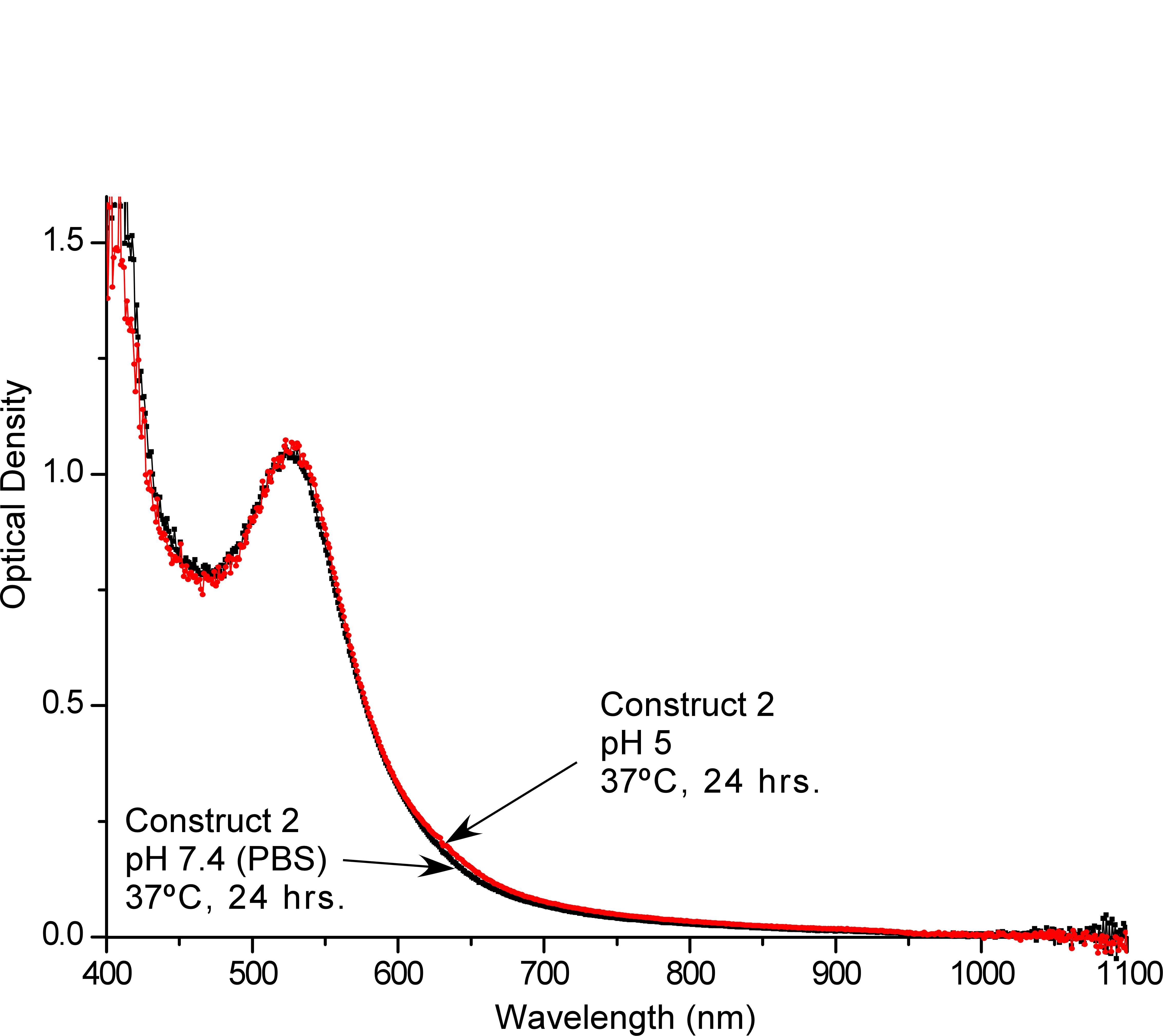

Supplement: Figure S9 — Spectra of Construct 2 incubated at pH 5 for 24 hrs at 37°C (red) showed no change from incubation at pH 7.4 (black). (TIF) [file pone.0088414.s009.tif]

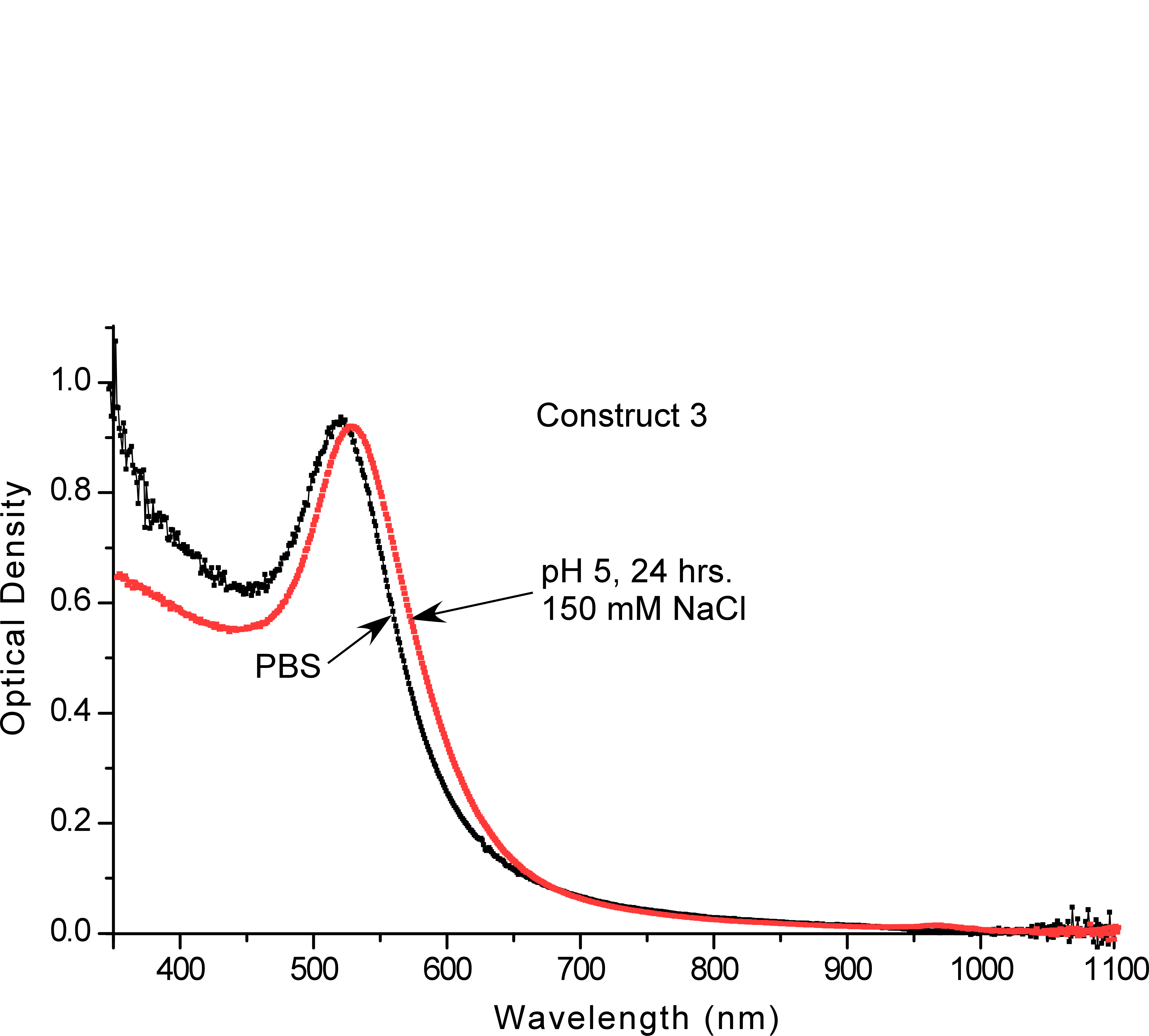

Supplement: Figure S10 — Spectra of Construct 3 incubated at pH 5 for 24 hrs at 37°C (red) showed slight change from incubation at pH 7.4 (black). (TIF) [file pone.0088414.s010.tif]

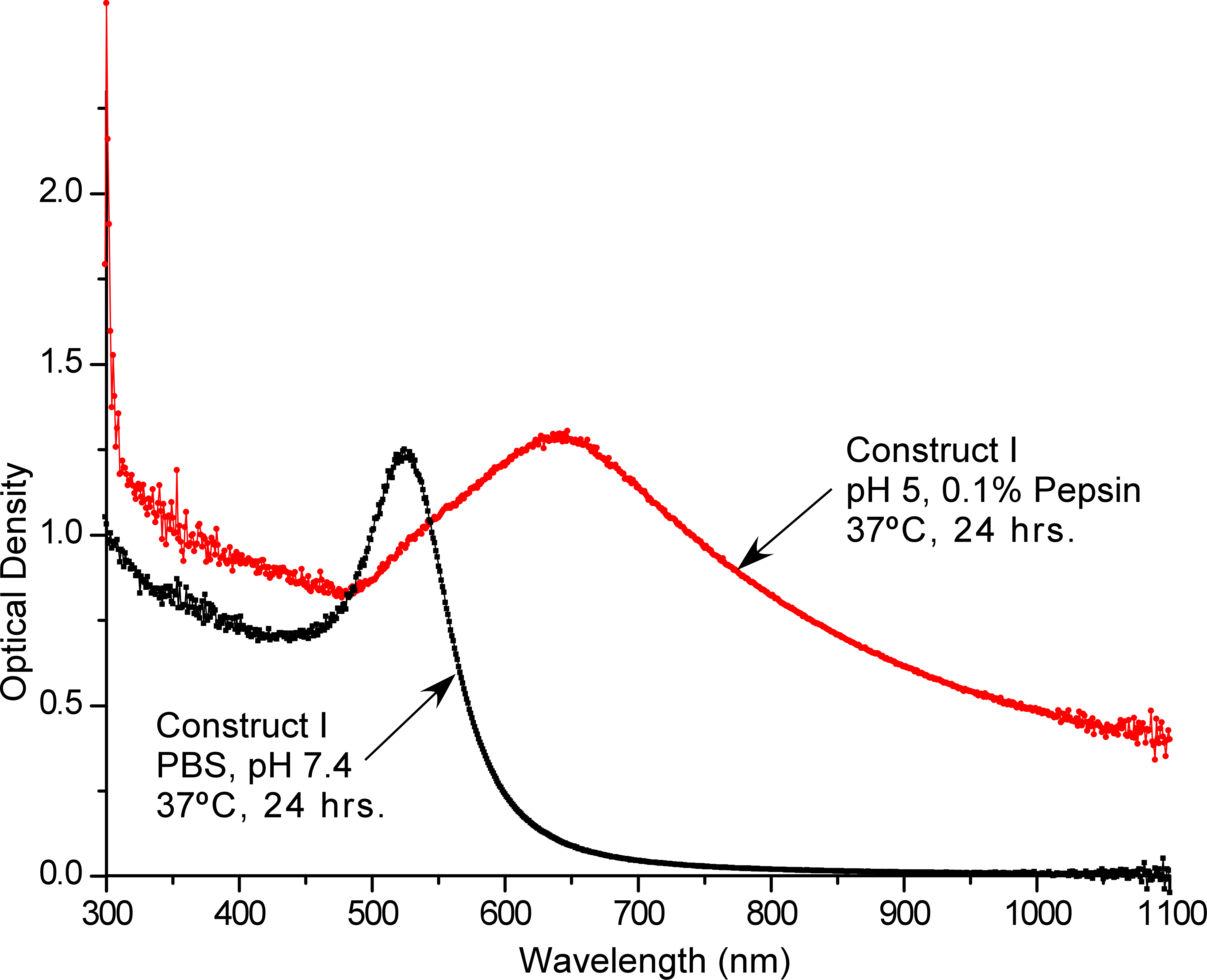

Supplement: Figure S11 — Shift of absorption into the NIR region when Construct 1 (black) was exposed to pepsin (red) for 24 hrs at pH 5. (TIF) [file pone.0088414.s011.tif]

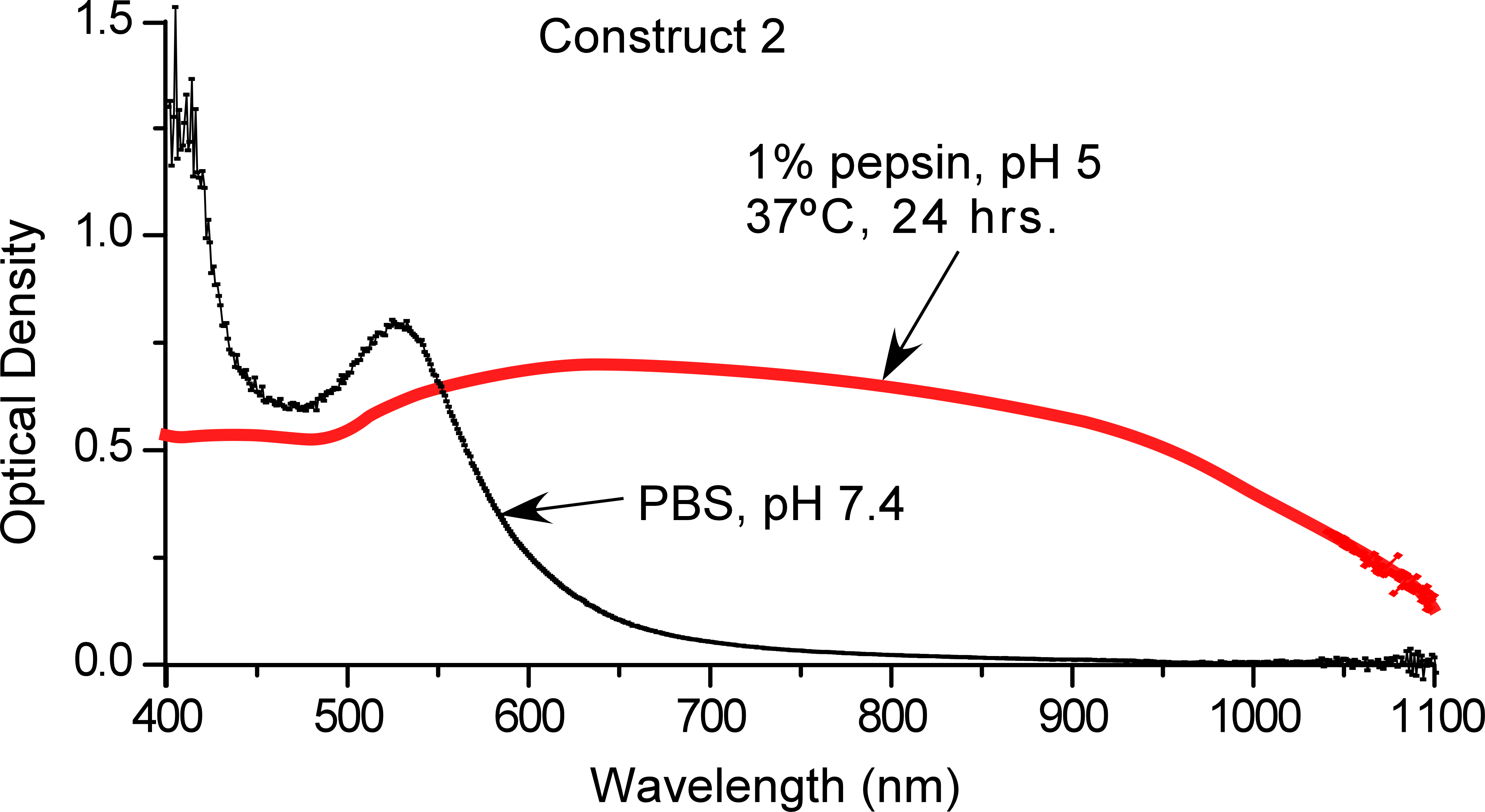

Supplement: Figure S12 — Shift of absorption into the NIR region when Construct 2 (black) was exposed to pepsin (red) for 24 hrs at pH 5. (TIF) [file pone.0088414.s012.tif]

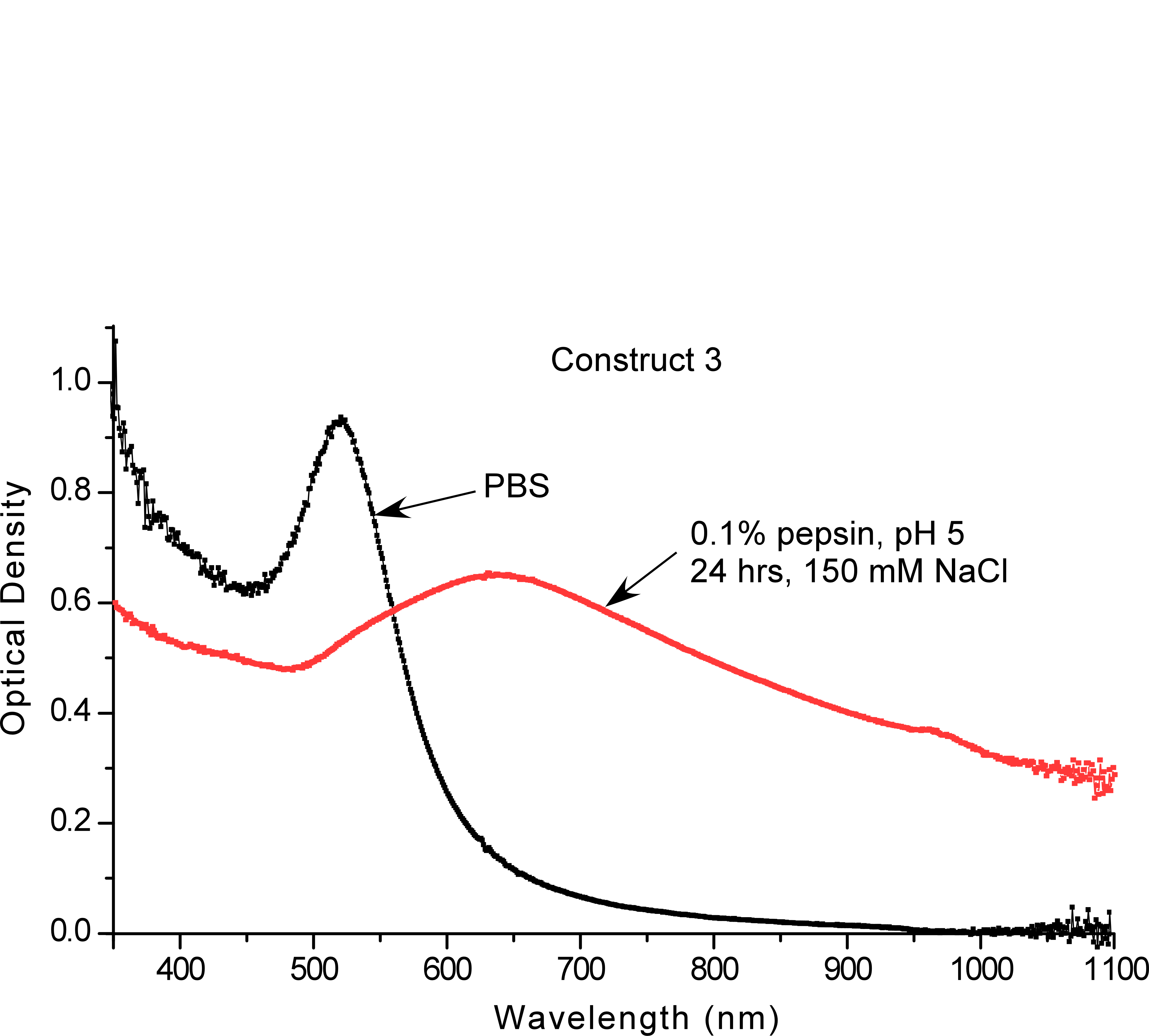

Supplement: Figure S13 — Shift of absorption into the NIR region when Construct 3 (black) was exposed to pepsin (red) for 24 hrs at pH 5. (TIF) [file pone.0088414.s013.tif]

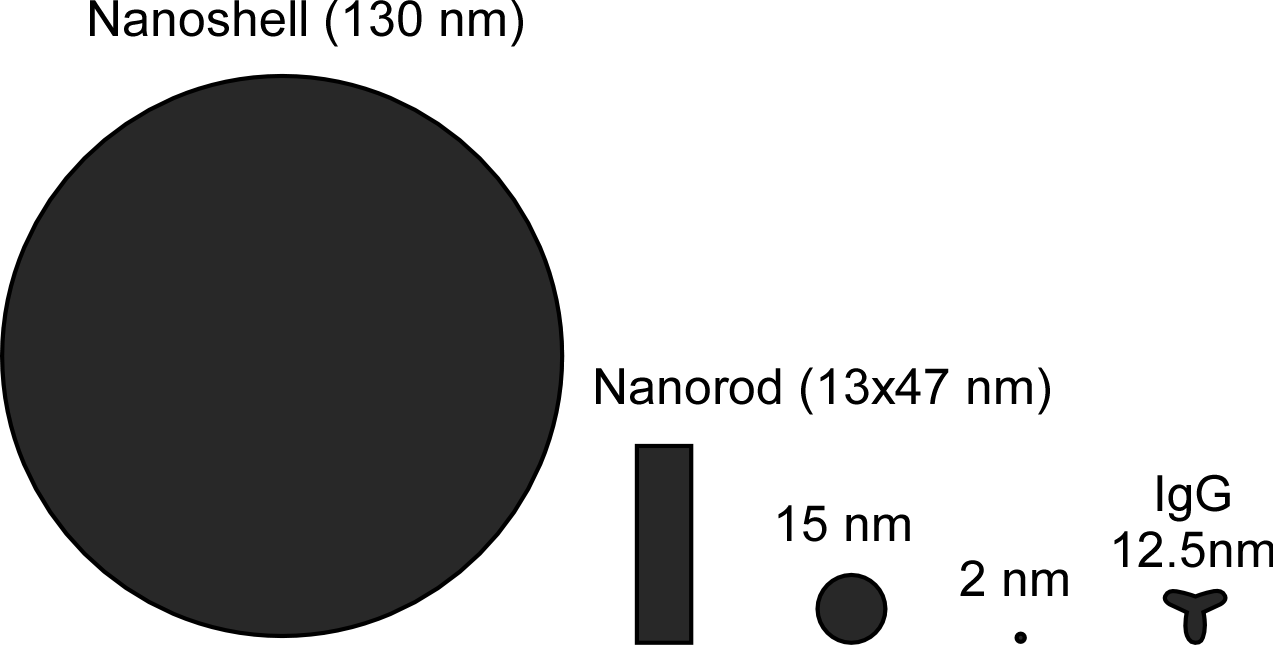

Supplement: Figure S14 — Size comparison of gold nanoshells, nanorods, 15 and 2 nm gold particles and an IgG molecule. The smaller ones may have substantially better diffusion, tumor access, and clearance properties. (TIF) [file pone.0088414.s014.tif]

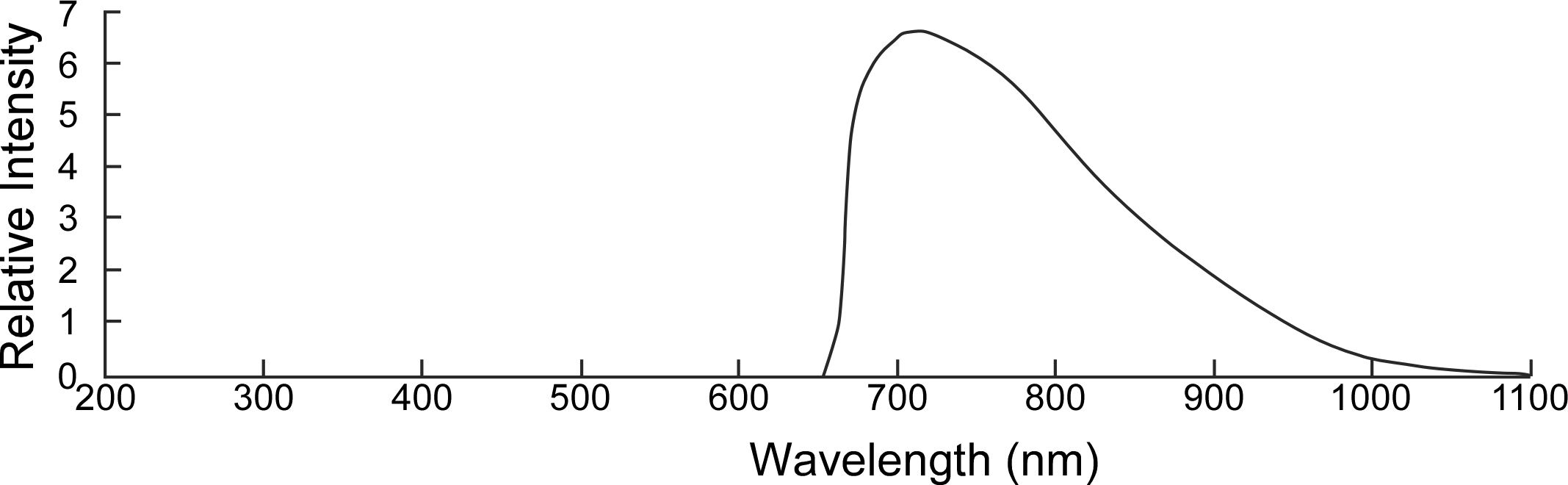

Supplement: Figure S15 — Water filtered infrared A (wIRA) lamp spectrum used with 665 nm pass filter. (TIF) [file pone.0088414.s015.tif]
