# Supplementary material for: Infrared-Transparent Gold Nanoparticles Converted by Tumors to Infrared Absorbers Cure Tumors in Mice by Photothermal Therapy
Source: PLoS One. 2014 Feb 10;9(2):e88414. doi: 10.1371/journal.pone.0088414 (PMC3919775; doi:10.1371/journal.pone.0088414)
Supplement: File S1 — Supporting Information. (DOCX) [file pone.0088414.s016.docx]

**Supporting Information**

The constructs were tested under various conditions varying salt, pH, temperature, serum, albumin, and proteolytic enzymes to study their stability against aggregation until in the endosome/lysosomes (pH5 plus proteolytic enzymes). The conditions used were designed to mimic blood and extracellular environments. The desired property was to avoid aggregation before cell uptake so that the nanoparticles would not: a) become large and reduce tumor delivery and penetration, and b) become NIR absorbing until inside the tumor cells.

Dynamic light scattering was used to measure the hydrodynamic size of the constructs under the various test conditions (Table 1). UV-Vis spectroscopy was used to detect any aggregation-induced red shift in absorption into the NIR region, particularly at ~800 nm.

One test was exposure to and incubation with 90% (by volume) serum (fetal bovine serum) for 24 hrs at 37ºC. Results are shown in Figures S1-3. No shift in absorbance into the NIR region was detected, providing evidence that there would probably be no aggregation in the extracellular environment.

The constructs were also incubated with 5% BSA at 37ºC and showed no sign of aggregation (Figures S4-S7).

Incubation at pH 5 caused some aggregation. Results with Construct I are shown in Figure S8. Construct 2 showed no change when the pH was changed from 7.4 to 5.0 (Figure S9). Construct 3 showed a slight red shift after incubation at pH 5.0 (Figure S10).

Addition of pepsin at pH5 caused significant aggregation, as seen by the red shift of the plasmon peak, red shift of the absorption spectrum, and absorption in the NIR region (Figures 11-13).
